# Rational assembly of 3D network materials and electronics through tensile buckling

**DOI:** 10.1126/sciadv.adz0718

**Published:** 2025-09-10

**Authors:** Xiaonan Hu, Zhi Liu, Zhenjia Tang, Shiwei Xu, Zhangming Shen, Yue Xiao, Youzhou Yang, Renheng Bo, Shuheng Wang, Wenbo Pang, Yihui Zhang

**Affiliations:** ^1^ Applied Mechanics Laboratory, Department of Engineering Mechanics, Tsinghua University, Beijing 100084, P.R. China.; ^2^ State Key Laboratory of Flexible Electronics Technology, Tsinghua University, Beijing 100084, P.R. China.; ^3^ Mechano-X Institute, Department of Engineering Mechanics, Tsinghua University, Beijing 100084, P.R. China.

## Abstract

Bioinspired network designs are widely exploited in biointegrated electronics and tissue engineering because of their high stretchability, imperfection insensitivity, high permeability, and biomimetic J-shaped stress-strain responses. However, the fabrication of three-dimensionally (3D) architected electronic devices with ordered constructions of network microstructures remains challenging. Here, we introduce the tensile buckling of stacked multilayer precursors as a unique route to 3D network materials with regularly distributed 3D microstructures. A data-driven topology optimization framework enables efficient search of the optimal 2D precursor pattern that maximizes out-of-plane dimension of the resulting 3D network material. Computational and experimental results demonstrate rational assembly of optimal multilayer precursor structures into well-architected 3D network materials with an evident interlayer separation. The resulting 3D network materials offer anisotropic, tunable J-shaped stress-strain curves, which can be tailored to reproduce stress-strain responses of biological tissues. Demonstration of reconfigurable volumetric 3D display suggests rich application opportunities in biointegrated electronics and tissue scaffolds.

## Teaser

Tensile buckling of stacked multilayers provides a route to the rational assembly of 3D network materials and electronics.

## INTRODUCTION

Soft biological tissues (e.g., skin, vessel, and muscle) are usually composed of differently oriented wavy collagen fibers that are connected at certain joints, forming a structural support that can undergo mechanical loadings along various directions (*1*, *2*). Inspired by the wavy network construction, artificial network materials consisting of two-dimensional (2D) or 3D curved fiber microstructures have been developed to reproduce nonlinear mechanical responses of soft biological tissues along certain or multiple directions (*3*–*7*). Owing to the low modulus (*8*), high stretchability (*9*), imperfection insensitivity (*10*–*12*), and biomimetic J-shaped stress-strain responses (*13*, *14*), these network designs have been widely exploited in the development of tissue scaffolds (*15*–*21*), implanted therapeutic devices (*22*–*25*), and skin-integrated electronics (*26*–*32*). While network materials with 3D micro-architectures offer increased design freedom than their 2D counterparts and improved capabilities to mimic anisotropic mechanical responses of biological tissues, their manufacturing remains a challenge (*33*), especially when the 3D network is made of high-performance electronic materials. For example, both nozzle-based (*34*–*36*) and light-based (*37*–*39*) 3D printing techniques have been exploited to fabricate 3D network materials, but these techniques are incapable of incorporating complex electronic circuits into printed 3D network materials.

Mechanically guided 3D assembly methods (*40*–*43*) rely on controlled deformations to transform patterned planar structures into desired 3D configurations, which are compatible with well-established microfabrication techniques, thereby providing a potential solution to address the manufacturing of 3D network structures in inorganic electronic materials. However, existing studies on the mechanically guided 3D assembly mostly focused on the assembly of single-layer structure (*44*). Although two previous publications reported the assembly of 3D mesostructures from releasable multilayer structures (*45*, *46*), they lack periodicity in the height direction because of the boundary effect induced by the elastomeric substrate. Because of the challenge in achieving reliable layer-by-layer propagation of out-of-plane deformations, it remains elusive to construct 3D network materials using mechanically guided 3D assembly methods.

In this work, we report the assembly of 3D network materials through tensile buckling of stacked multilayer precursors, where each precursor layer consists of periodically distributed constructions of 2D ribbon structures. Instead of using an elastomer substrate to apply forces in traditional assembly methods (*47*, *48*), we apply uniaxial tensile forces directly to the two ends of stacked multilayer precursors to ensure relatively uniform driving forces of tensile buckling and, consequently, relatively uniform out-of-plane buckling displacements across different precursor layers. We develop a data-driven topology optimization framework that allows efficient search of the optimal 2D precursor pattern to maximize the out-of-plane displacement during buckling assembly. The framework relies on finite element analyses (FEAs) to solve the highly nonlinear postbuckling problem with consideration of interlayer contact and exploits the differential evolution algorithm to search for the optimal solution. The optimal multilayer precursor structures from the design optimization can be assembled into well-architected 3D network materials with an evident interlayer separation (390 μm, in relative to the in-plane dimension of 325 μm by 700 μm for a basic unit cell), as validated by assembly experiments using polyimide (PI) as the constituent material. The fabricated 3D network materials offer anisotropic J-shaped stress-strain curves with a high degree of tunability, which can be also tailored to reproduce stress-strain responses of soft tissues (e.g., pig thoracic aorta). The demonstration of reconfigurable volumetric 3D display shows the capability of the proposed method to incorporate inorganic electronic components into the 3D network architecture, suggesting promising applications in flexible electronic devices.

## RESULTS

### Mechanically guided assembly of 3D network materials

**Fig. 1.**
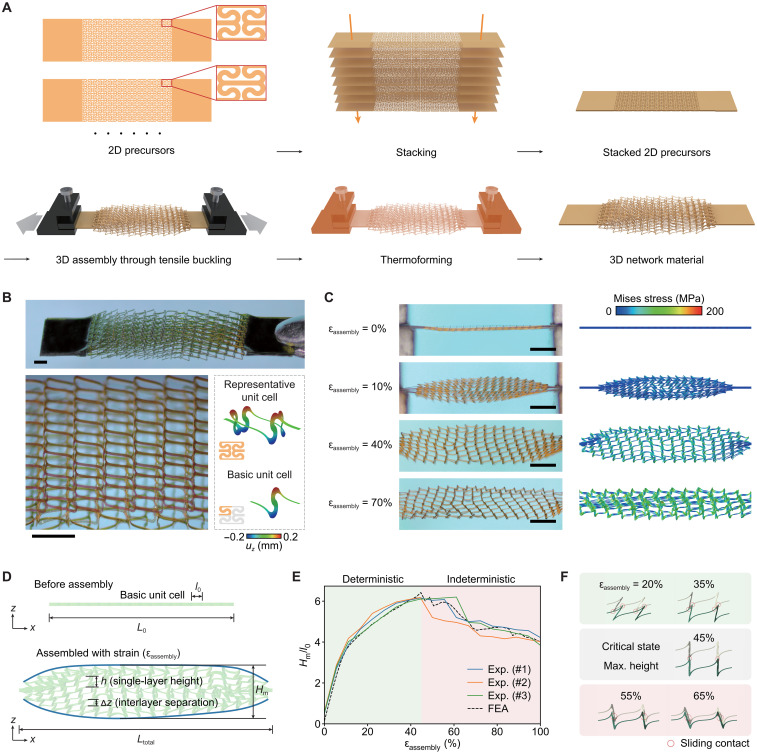
Conceptual illustration of the mechanically guided assembly of 3D network materials. (**A**) Schematic illustration of the formation process of 3D network materials through tensile buckling of stacked 2D multilayer precursors. (**B**) Optical image of the as-fabricated 3D network material and a magnified view of the periodic network. Scale bars, 1 mm. (**C**) Optical images and corresponding results of FEAs on the deformation sequences during the tensile buckling. Scale bars, 2 mm. (**D**) Definition of key geometric features of the network material before and after assembly. (**E**) Normalized height of the multilayer sample versus the assembly strain, where the deterministic and indeterministic stages are marked with different background colors. Here, experimental results of three separate samples are shown, along with FEA results. (**F**) Evolution of geometric configuration for representative unit cells in the sample that highlight the change of interlayer contact mode.

Figure 1A illustrates the concept of harnessing tensile buckling of stacked planar multilayers for mechanical assembly of 3D network materials. Photolithographic techniques allow patterning of each precursor layer (e.g., PI with 10 μm in thickness) with desired 2D periodic lattice construction. Stacking them together forms a multilayer precursor structure with sliding interfaces, which is then clamped at two ends by a mechanical stage. Uniaxial stretching triggers lateral buckling in the multilayer precursor structure because out-of-plane bending and twisting deformations are more energy favorable in thin ribbon microstructures (100 μm in width and 10 μm in thickness) of each precursor layer. By using the shape memory effect, these out-of-plane deformations can be memorized and retained after a temperature cycle (i.e., heating up to 220°C and cooling down to room temperature). Consequently, a 3D network material with well-architected curvy ribbon microstructures can be formed. Figure 1B presents optical images of a 3D network material fabricated using this mechanical assembly method, where an eight-layer precursor structure (see fig. S1 for details) and a tensile strain (ε_assembly_ = 40%) were adopted in the assembly. Because of the periodicity of microstructures in each precursor layer, the out-of-plane displacements are relatively uniform in the middle region of the sample, noting that the boundary effect does exist in the adjacent region of the two ends (see texts S1 and S2 and figs. S2 and S3 for details on boundary constraints). A basic unit cell of the 3D network has a height of 390 μm and a lateral dimension of ~325 μm by 700 μm, where a basic unit cell is referred to as one-fourth of a representative unit cell, considering the mirror symmetry of the geometry (Fig. 1B). Although the curvy ribbon microstructure in the network has two different possible buckling modes, their resulting 3D configurations have similar heights and mechanical responses (fig. S4). As a result, the entire 3D network has a relatively uniform height (fig. S5) and a well-controlled stress-strain curve after the formation. Furthermore, the proposed mechanically guided assembly method is applicable to a range of different constituent materials, such as PI (Fig. 1B), polyethylene terephthalate (PET; fig. S6) and metal/polymer laminate to be discussed in the application section.

Figure 1C and movies S1 and S2 show the dynamic assembly process and optical images of a multilayer network sample. Mechanical modeling based on FEA (see Materials and Methods for details) well captures coupled bending and twisting deformations of ribbon components in the network, as evidenced by the reasonable agreement of deformed configurations. In this assembly method, the accessible height of assembled 3D network structure (along the out-of-plane direction) corresponds to an important metric of the fabrication method. Basically, the total height (*H*_m_) of the entire 3D network can be approximately calculated by *H*_m_ = (*n* − 1)Δ*z* + *h*, where *n* is the number of layers, Δ*z* is the interlayer separation, and *h* is the single-layer height after the assembly (Fig. 1D). In the following analyses, the in-plane size (*l*_0_) of the basic unit cell (before assembly) is used to normalize the single-layer height, interlayer separation, and total height. Figure 1E shows the dependence of the normalized total height (*H*_m_/*l*_0_) on the assembly strain (ε_assembly_) for the design described in Fig. 1 (B and C). The deformations induced by the tensile buckling can be categorized into two stages. In the first stage (ε_assembly_ below ~45%), the total height increases monotonously with increasing the assembly strain because the contact points move continuously in the corresponding ribbons, without any switch of contact modes (Fig. 1F). Consequently, the total height of 3D network material can be deterministically controlled by the assembly strain in the first stage (also termed as deterministic stage). Above a critical strain (~45%), the deformation enters the second stage (i.e., indeterministic stage) because the contact state between the ribbons of adjacent layers begins to switch (Fig. 1F) in certain unit cells, resulting in a collapse and a reduction of the entire height. Because the collapse occurs in a random manner across the various unit cells of the entire network, a certain degree of discrepancy among various network samples can be observed in Fig. 1E, indicating that the total height could not be deterministically controlled in this stage. Therefore, an assembly strain below the critical strain (~45%) should be adopted to allow a reliable control of the microstructure geometry and height. According to the FEA and experimental results, the maximum total height of the entire 3D network is around 6.2 times larger than that of the in-plane size (*l*_0_) of the basic unit cell.

**Fig. 2.**
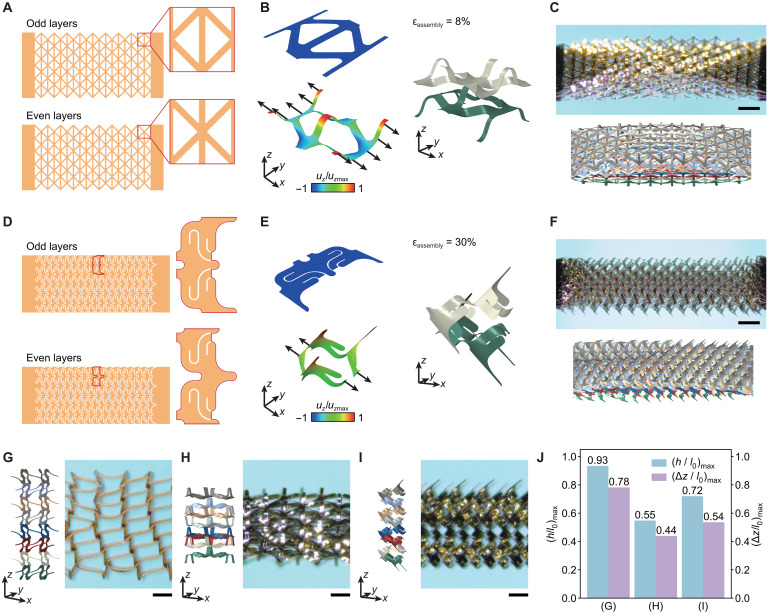
Comparison of 3D network materials assembled from 2D multilayer precursors with different designs. (**A**) Illustration of alternating multilayer precursors, each with a fully triangular lattice design. (**B**) Undeformed and deformed configurations of a representative unit cell in a single layer presented in (A), along with the stacked configuration. (**C**) Optical image and the corresponding FEA result of the as-fabricated network material based on the design shown in (A). Scale bars, 1 mm. (**D**) Illustration of alternating multilayer precursors, each with a periodic kirigami design. (**E**) Undeformed and deformed configurations of a representative unit cell in a single layer presented in (D), along with the stacked configuration. (**F**) Optical image and the corresponding FEA result of the as-fabricated network material based on the design shown in (D). Scale bar, 1 mm. (**G** to **I**) Cross-sectional view of 3D architected materials assembled from 2D multilayer precursors presented in Fig. 1, in (A), and in (D). Scale bars, 0.5 mm. (**J**) Comparison of the normalized single-layer height and interlayer separation among the three assembled architected materials presented in (G) to (I).

The above results suggest that the thin multilayer network precursor (80 μm in total thickness for the design in Fig. 1B) can be transformed into an evident 3D construction with a thickness (or height) of 3.1 mm, around 39 times of the initial thickness, based on the proposed tensile buckling strategy. To quantify the height change resulted from the assembly, the maximum accessible normalized interlayer separation (Δ*z*/*l*_0_)_max_ that can be achieved before the failure of the constituent material is introduced to characterize the out-of-plane deformability of a certain multilayer network design. From the perspective of 3D fabrication, this out-of-plane deformability represents a key metric to consider. In general, the maximum accessible normalized interlayer separation depends highly on the multilayer network design. For example, Fig. 2 and figs. S7 and S8 present a comparison of three typical designs, including the aforementioned design, a fully triangular lattice design, and a kirigami design inspired by snake skin (*49*, *50*). Distinct geometries of 3D micro-architectures can be formed based on those designs. In particular, the different layers of the 3D network material in Fig. 2C are separated by arch-shaped ribbons (along the *y*-axis direction) formed during the assembly process. The tensile buckling of kirigami-based precursors (Fig. 2F) induces ordered spatial rotation of the spikes, leading to the formation of a snake skin–like 3D configuration. The corresponding values of maximum accessible normalized interlayer separation are provided in Fig. 2J, along with values of maximum accessible normalized single-layer height. A clear difference of (Δ*z*/*l*_0_)_max_ (0.78 versus 0.44) can be observed, suggesting the importance of carrying out a topology optimization of multilayer precursor pattern to yield the largest (Δ*z*/*l*_0_)_max_.

### Topology optimization of the multilayer precursor design

The tensile buckling of multilayered planar films involves nonlinear postbuckling deformations, contact with substantial sliding, and complex bifurcation paths, corresponding to a mechanics problem with a high degree of complexity (*51*–*54*). While previous works have addressed the topology optimization of multiple deformable bodies in contact with large deformations (*55*–*58*), they primarily focused on 2D problems or simple 3D block contacts, which are not applicable to solving the problem studied here. The specific challenges in this study include, (i) unlike traditional objectives such as minimizing strain energy or weight, there is no established criterion for maximizing out-of-plane deformation of planar shells, and (ii) handling contact between shells is challenging due to various contact forms (e.g., surface-to-surface, edge-to-surface, and edge-to-edge contacts), necessitating the use of data-driven methods, which are particularly well-suited for complex nonlinear problems (*59*–*68*).

In this study, we introduce a data-driven topology optimization framework that relies on explicit FEA with speeding-up strategies to solve the forward problem efficiently (see Materials and Methods for details) and exploits the differential evolution algorithm to search for the optimal solution. Note that the interlayer separation of assembled 3D network materials is highly relevant to the out-of-plane displacement resulted from the tensile buckling of a single-layer precursor. The design optimization of a single-layer network structure is much simpler than that of multilayer counterpart because it does not involve the interlayer contact and requires much less elements in the FEA. Based on these considerations, we first carry out the topology optimization of a single-layer network structure to yield the optimal geometric feature that gives the largest single-layer height and then take into account the interlayer contact to determine the optimal design of multilayer network precursor.

**Fig. 3.**
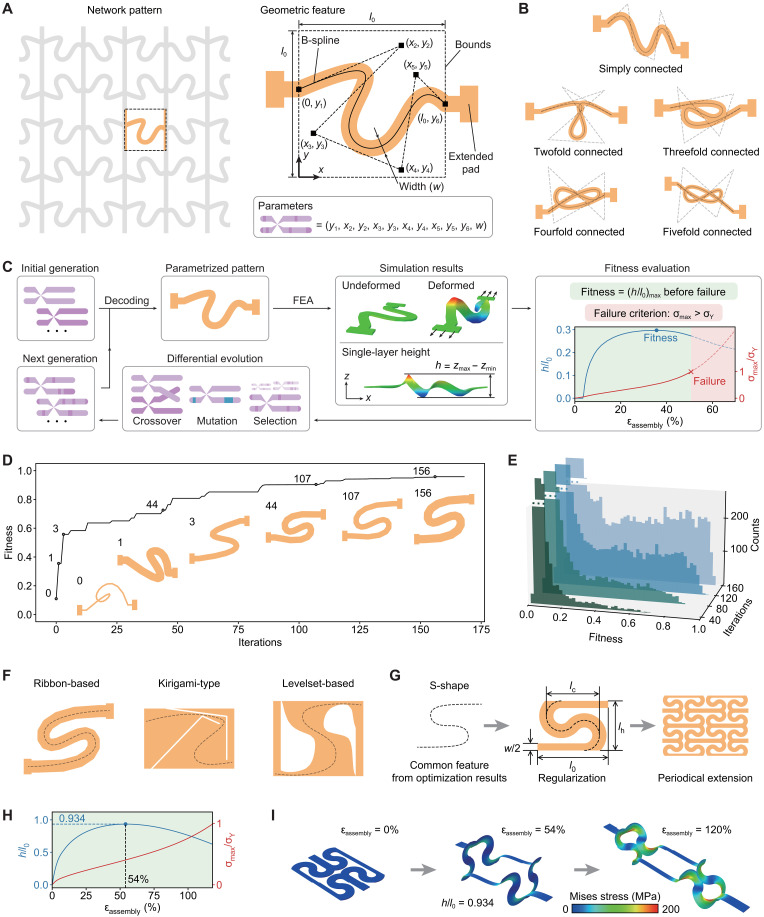
Data-driven topology optimization of the single-layer 2D network precursor. (**A**) Illustration of the network pattern and a representative geometric feature, along with the parametrization scheme for a curved ribbon used in the topology optimization. (**B**) Schematic flow chart of the topology optimization based on differential evolution algorithm and FEA. (**C**) Definition of the objective function (i.e., maximum normalized height before failure) in the topology optimization. (**D**) Fitness evolution curve of the topology optimization, where representative intermediate configurations during iterations are presented. (**E**) Set of histograms of the fitness of individuals accumulated to different stages of iteration. (**F**) Optimization results based on three parametrization schemes, including ribbon-based, kirigami-type, and level set–based schemes. Geometric features of these three patterns are highlighted with dashed lines. (**G**) Construction of a periodic 2D network pattern based on common features extracted from the optimization results. The key geometrical parameters of the basic unit cell are labeled in the middle panel. (**H**) Normalized height and maximum normalized Mises stress of the optimized representative unit cell versus the assembly strain. (**I**) Undeformed and deformed configurations (ε_assembly_ = 0, 54, and 120%) of the optimized representative unit cell, along with the distribution of the Mises stress.

Figure 3A presents a typical 2D network pattern consisting of periodical constructions of curved and straight ribbons. Because the geometric feature that contributes to the out-of-plane displacement is usually the curved ribbon whose end-to-end connection basically aligns with the loading direction, we focus on the topology optimization of this curved ribbon, as shown in the right panel of Fig. 3A. The middle line of the curved ribbon is characterized by the B-spline curve that can be captured using a series of controlling points (*x_i_*, *y_i_*) (*i* = 1,…, *m*). Those controlling points are restricted in a rectangular region, such that the resulting B-spline is also located within this rectangular region. The width of the curved ribbon is denoted by *w*. This modeling method allows the generation of planar ribbon patterns with a diverse set of topologies (e.g., those that are threefold, fourfold or fivefold connected) for a small number (e.g., *m* = 6) of controlling points (Fig. 3B). Figure 3C presents the flowchart of the topology optimization based on differential evolution algorithm, noting that this algorithm is very suitable for solving the global optimization problem and is compatible with parallel computation. The optimization parameters are the coordinates of all controlling points and the ribbon width. The optimization objective (i.e., fitness in differential evolution algorithm) is the maximum normalized out-of-plane displacement (*h*/*l*_0_)_max_ of the structure that can be achieved before the failure of the constituent material. Here, PI serves as the constituent material in the optimization, and the Mises stress criterion is adopted to evaluate the plastic yielding. Specifically, the failure occurs when the maximum Mises stress in the entire structure reaches the yield point (σ_Y_ = 200 MPa for PI). Note that the thickness of the precursor is not treated as an optimization parameter, as it does not significantly affect the maximum out-of-plane deformation in the condition that it is not excessively large and does not cause early failure (see text S3 and fig. S9 for details). Figure 3D and fig. S10 present the results of topology optimization by using randomly created ribbon patterns as the initial generation, where *m* = 6 is adopted. The fitness increases rapidly during the first 25 iterations and converges to ~0.96 after 168 iterations. Figure 3E shows the fitness distribution for all the history individuals generated after 40, 80, 120, and 160 iterations. After 160 iterations, a steep descending edge can be observed at the highest fitness level, indicating that further improvement of the fitness is extremely challenging. In view of the global exploration capabilities of differential evolution algorithms, such a steep descending edge suggests that the optimization process has approximately reached the global optimal. The optimal pattern resulted from the topology optimization is an S-shaped ribbon shown in the left panel of Fig. 3F. Notably, if a kirigami pattern (or a levelset-based scheme) (figs. S11 to S14) is adopted to describe the planar topology instead of a curved ribbon, the S-shaped structure still corresponds to the optimal pattern that maximizes the out-of-plane displacement before material failure (Fig. 3F). These results suggest that the S-shaped pattern represents a key geometric feature to yield a large height through the tensile buckling. Consequently, a periodic network construction can be obtained through mirroring of the S-shaped pattern and periodical extensions along two orthotropic directions (Fig. 3G and text S4). Such a network structure reaches the peak out-of-plane displacement [(*h*/*l*_0_)_max_ = 0.934] at ε_assembly_ = 54% (Fig. 3, H and I). In addition, the material failure occurs when the assembly strain is above 120%, and the failure regions with the maximum Mises stress are in accordance with the experimental observations (fig. S15).

**Fig. 4.**
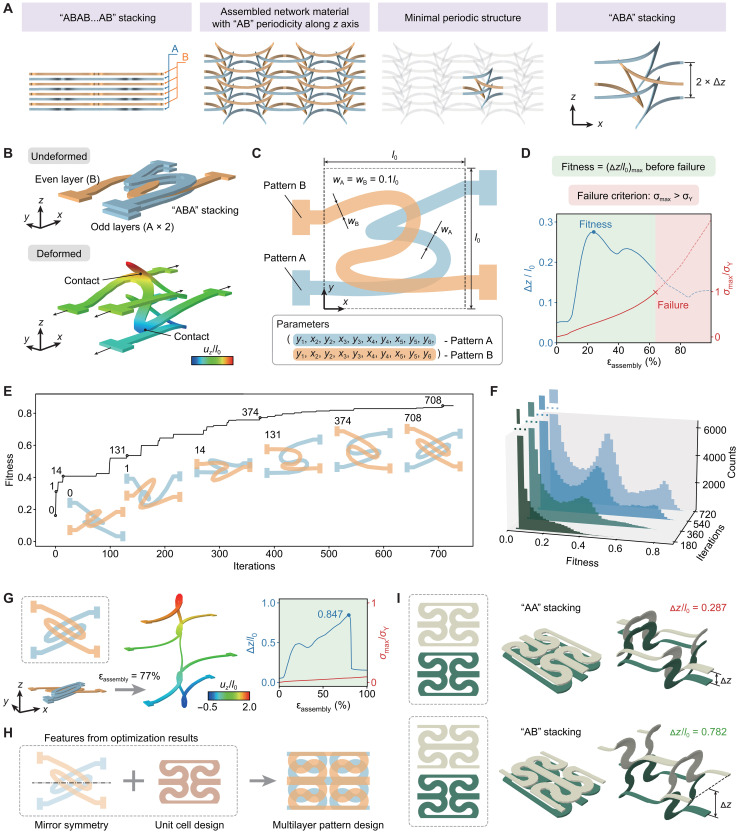
Data-driven topology optimization of the multilayer 2D network precursor considering the interlayer interaction. (**A**) Parametrization scheme for stacked multiple layers of ribbon-based 2D precursor patterns used for topology optimization. (**B**) Illustration of the ABA stacking of different layers and the configuration of a typical multilayer design before and after assembly, featuring the interlayer separation. (**C**) Illustration of the parametrization scheme used in the topology optimization. (**D**) Definition of the objective function (i.e., maximum normalized interlayer separation before failure) in the topology optimization. (**E**) Fitness evolution curve of the topology optimization, where representative intermediate configurations during iterations are presented. (**F**) Set of histograms of the fitness of individuals accumulated to different stages of iteration. (**G**) Optimal multilayer precursor design, the resulting 3D configuration, and the relationship between interlayer separation and assembly strain. (**H**) Construction of a periodically stacked network pattern based on geometric features obtained from the topology optimization. (**I**) Comparison of interlayer separation between two stacking strategies.

The topology optimization of the multilayer film structure is much more complex than that of the single-layer structure due to the interlayer contact and the substantially increased number of optimization parameters. To simplify the problem, we consider a multilayer design periodically distributed along the thickness direction (Fig. 4A), with the stacking order of “ABAB…AB,” where “A” and “B” denote two network layers with different 2D patterns. To model these periodically stacked network structures, a three-layer construction (“ABA”) can be adopted for simplicity (Fig. 4B). Following the strategy discussed in Fig. 3, both patterns of A and B layers are represented by curved ribbons whose middle lines are characterized by B-spline curves (Fig. 4C). In this case, the ribbon widths of both layers are fixed as *w*_A_ = *w*_B_ = 0.1 *l*_0_, noting that the ribbon width plays a relative minor role on the interlayer separation, in comparison to the geometric configuration. Because the ribbon geometry of each layer is decided by separate controlling points, there are (2*m* − 2) optimization parameters in total. The optimization objective (i.e., fitness in differential evolution algorithm) is the maximum normalized interlayer separation (Δ*z*/*l*_0_)_max_ that can be achieved before the failure of the constituent material (Fig. 4D).

Figure 4E provides the results of topology optimization by using randomly created ribbon patterns as the initial generation, where *m* = 6 is adopted. Movie S3 visualizes the optimization process by showing the evolution of the precursor patterns throughout the iterations. In comparison to the optimization of single-layer structure, the current optimization converges much more slowly (727 Iterations versus 168 iterations) (table S1). The convergence involves FEA calculations of more than 200,000 individuals, highlighting the importance of data size on the topology optimization of multilayer network structures. Figure 4F shows the fitness distribution during the iterations. The steep descending edge at the highest level of the histogram after 720 iterations suggests that the global optimal solution has been reached. Specifically, most of the individuals generated during the first 180 iterations show relatively poor performance, as evidenced by the fitness distribution (i.e., very few individuals give the fitness above 0.5). After 360 iterations, a local peak can be observed at the middle section of the histogram, which is different from the case of single-layer structure (Fig. 3E). This suggests that more local optimal points exist in the optimization problem of multilayer structure, showing the increased complexity than that of single-layer structure.

Figure 4G shows the optimal multilayer pattern resulted from topology optimization, featuring tilted S-shaped patterns for both layers. The S-shaped ribbons in these two layers show a mirror symmetry, such that the out-of-plane deformation can be well supported by each other, leading to a maximized interlayer separation (Δ*z*/*l*_0_ = 0.847). This mirror symmetry can also be observed when applying a level set–based parametrization scheme to optimize the multilayer precursor patterns (figs. S16 and S17). It is also noteworthy that the optimized ribbon pattern in each layer is very similar to the S-shaped pattern in Fig. 3F.

The S-shaped pattern and the mirror symmetry between the A and B layers serve as critical geometric features for achieving substantial 3D network height. Under tensile loading, the central segment of the S-shaped ribbon undergoes large rotation, driving substantial out-of-plane displacement of each layer. The mirror-symmetric design ensures reliable supporting structures between adjacent layers, enabling the accumulation of out-of-plane displacement throughout all layers. Notably, these geometric characteristics still maintain, when the optimization objective function is redefined as Δ*z*/*l* (normalized by the deformed unit cell length *l* rather than the initial length *l*_0_) to characterize the aspect ratio in the *x*-*z* plane (fig. S18). The optimization process consistently yields geometrically analogous results, highlighting the importance of these design features.

Based on the mirror symmetry of ribbon patterns in adjacent layers and the periodical pattern obtained in Fig. 3G, a stacked multilayer construction is proposed, as described in the right panel of Fig. 4H (see text S4 for details). The 3D network structure assembled from this slightly modified design offers an enhanced out-of-plane loading capability under compression in comparison to that from the design optimization. Because of the finite boundary effect, the top and bottom layers of the multilayer sample are stretched more than the middle layers (Fig. 1C), such that the assembled 3D network structures in the middle layers are subject to a certain degree of out-of-plane compression. Therefore, an improved out-of-plane compressive loading capability is beneficial for maintaining a large interlayer separation in practical conditions, especially for designs with a large number (e.g., >6) of layers. Based on the proposed multilayer design, the resulting network structure reaches a peak interlayer separation of [(Δ*z*/*l*_0_)_max_ = 0.782] at ε_assembly_ = 45% (Fig. 4I), which is much larger than the case of simple stacking (without alternation) (fig. S19).

### Nonlinear mechanical responses with a high degree of tunability

**Fig. 5.**
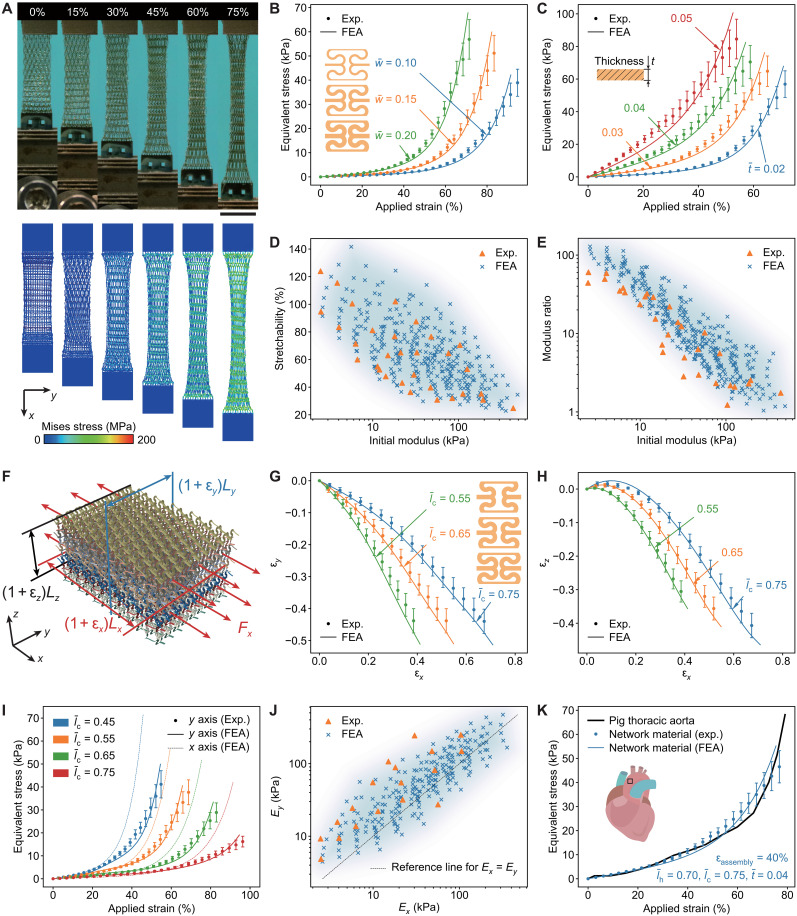
Tunable nonlinear mechanical responses of the 3D network material. (**A**) Optical images the 3D network material under different levels of uniaxial stretching, and the corresponding FEA results. Scale bar, 5 mm. (**B** and **C**) Effects of two key geometric parameters (normalized width $\bar{w}=w\;/\;l_{0}$ and normalized thickness $\bar{t}=t\;/\;l_{0}$ ) on the J-shaped uniaxial stress-strain curves of the 3D network materials. (**D** and **E**) Feasible region for tuning the uniaxial mechanical response of the 3D network material in terms of initial modulus/stretchability (D) and initial modulus/modulus ratio (E). The experimental and FEA results are marked as data points, and the background color represents the area covered by FEA results. (**F**) Definition of the coordinate system and strain components along three orthogonal directions for the 3D network material. (**G** and **H**) Transverse strains (ε*_y_* and ε*_z_*) versus the strain (ε*_x_*) along the stretching direction for 3D network materials with three different normalized effective lengths ( ${\bar{l}}_{c}=0.55,0.65\;\text{and}\;0.75$ ). (**I**) Uniaxial stress-strain curves along *x*- and *y*-axis directions for 3D network materials with a set of different normalized effective lengths ( ${\bar{l}}_{c}=0.55,0.65\;\text{and}\;0.75$ ). (**J**) Feasible region for tuning the two initial moduli (*E_x_* and *E_y_*) of the 3D network material. The black dotted line is a reference line indicating the same initial modulus along *x*- and *y*-axis directions. (**K**) Inverse design of the 3D network material whose uniaxial J-shaped stress-strain curve (along *x*-axis direction) matches that of a pig thoracic aorta.

The 3D network materials formed through the multilayer network design in Fig. 4H have highly tunable mechanical responses. The overall mechanical properties of the 3D network material can be characterized by the equivalent stress-strain curve, which are calculated using the nominal stress and nominal strain based on the assembled 3D configuration of the network material. Figure 5 (A and B) (orange curve) presents the uniaxial stress-strain curve and deformation sequence of a typical 3D network material made of PI, with *w*/*l*_0_ = 0.15, *t*/*l*_0_ = 0.02, *l*_c_ /*l*_0_ = 0.75, and ε_assembly_ = 40%. At the initial stage of stretching (e.g., ε_applied_ < 40%), the curved ribbon microstructures are governed by bending and twisting deformations, resulting in a low tangential modulus (below 5 kPa). As the stretching proceeds, the ribbon microstructures are gradually realigned with the loading direction through coordinated bending/twisting deformations and spatial rotations. When the applied strain increases to ~70%, the ribbon microstructures are mostly aligned along the loading direction, and therefore, the network material becomes stretching-dominated, as evidenced by the substantially increased tangential modulus (~160 kPa). Such a switch of deformation mode leads to a J-shaped stress-strain curve that has been observed in many soft biological tissues.

By tailoring the geometric parameters (e.g., *w*/*l*_0_, *t*/*l*_0_, and *l*_c_/*l*_0_) of the multilayer network precursor, the J-shaped stress-strain curve of the 3D network materials formed through the proposed tensile buckling strategy can be tuned in a wide range (Fig. 5, B and C, and fig. S20). For example, the sharpness (or nonlinearity) of the J-shaped stress-strain curve reduces significantly with the increase of the normalized thickness (*t*/*l*_0_) from 0.02 to 0.05. Such a sharpness can be quantitatively characterized by the modulus ratio (*E*_peak_/*E*_initial_), defined as the ratio of the peak tangential modulus (*E*_peak_) (before material failure) to the initial modulus (*E*_initial_) (fig. S21). For instance, the modulus ratio is 88.5 at *t*/*l*_0_ = 0.02 based on FEA, which is much larger than the modulus ratio (7.1) at *t*/*l*_0_ = 0.05 in Fig. 5C. Combined experimental and computational results (Fig. 5, D and E) show that the key mechanical properties of proposed 3D network materials—including initial modulus, stretchability, and modulus ratio—can be tuned in a broad range. Here, the constituent material is fixed as PI, and the design parameters are in the range of [0.1, 0.2] for *w*/*l*_0_, [0.02, 0.05] for *t*/*l*_0_, [0.45, 0.75] for *l*_c_/*l*_0_, and [20%, 40%] for ε_assembly_. In particular, the stretchability and initial modulus can be adjusted in the range of [25%, 124%] and [2.5 kPa, 440 kPa], respectively, while the modulus ratio can be tuned from 1.2 to 60, according to experimental measurements. For a fixed constituent material, it is difficult to increase the initial modulus and the modulus ratio, simultaneously. In comparison, the tradeoff between the stretchability and the initial modulus is not very evident. For instance, at an initial modulus of ~100 kPa, the stretchability can be still tuned from ~32 to ~70% based on experimental results. Repetitive loading/unloading tests show relatively stable mechanical responses (fig. S22). In all the above results, mechanics calculations based on FEA (see Materials and Methods for details) agree reasonably well with experimental measurements, suggesting FEA as a reliable tool for the design of proposed 3D network materials.

The Poisson effect of developed 3D network materials can be also adjusted by tailoring the normalized effective length (*l*_c_ /*l*_0_) of the network precursor. Figure 5 (F to H) shows transverse strains (ε*_y_* and ε*_z_*) during the uniaxial stretching along the *x* direction, where ε*_y_* and ε*_z_* were determined based on the deformations at the middle region of the network samples. A shrinkage along the *y* direction can be always observed during the *x*-directional stretching. Differently, an expansion along the *z* direction can be noted at the initial stage of the *x*-directional stretching, especially for the case of *l*_c_/*l*_0_ = 0.75 because the assembly strain (40%) used in the formation is a bit smaller than the strain (~45% in experiments) to induce the maximum total height during tensile buckling (Fig. 1E). For a relatively large applied strain (e.g., >20%), the transverse strain varies approximately linearly with the applied strain. Here, the initial tangential Poisson ratios (including ν*_xy_* and ν*_xz_*) can be tuned in a broad range of values by adjusting the design parameters (fig. S23).

The developed 3D network material also offers well-controlled J-shaped stress-strain curve under uniaxial stretching along the *y* direction. By using a specimen design with connections in the *y*-axis direction, the *y*-axial mechanical properties of the 3D network material can be effectively characterized (fig. S24). Figure 5I presents testing results and numerical simulations for a set of network materials with different normalized lengths (*l*_c_/*l*_0_), where *w*/*l*_0_, *t*/*l*_0_, and ε_assembly_ are fixed as 0.1, 0.02, and 40%, respectively. Effects of other parameters (normalized width and assembly strain) on the mechanical responses along the *y* direction are illustrated in fig. S25. These results show a clear anisotropy of nonlinear mechanical responses, but the initial modulus and the stretchability along the *y* direction are on the same order as those along the *x* direction. By tailoring geometric parameters, the degree of anisotropy can be tuned in a certain extent. For instance, Fig. 5J shows a feasible region of two initial moduli (*E_x_* and *E_y_*) that can be accessed, according to FEA and experimental measurements, which can serve as a design guideline for practical uses. Owing to the high porosity (~90%), the developed 3D network material shows excellent elastic responses under out-of-plane compression. After the 1st compression and release, the ribbon microstructures of the 3D network can mostly recover during further loading and unloading (e.g., with ~60% compressive strain) (fig. S26).

Because of the highly tunable J-shaped stress-strain curves, the developed 3D network material can be tailored to reproduce the nonlinear mechanical responses of soft biological tissues. Figure 5K presents an example, in which the stress-strain curve of the optimized 3D network agrees well with that of the design target [pig thoracic aorta (*69*)]. Besides, the 3D network materials formed through use of other precursor designs (e.g., the fully triangular lattice design and the kirigami design in Fig. 2) also provide stable J-shaped stress-strain curves under uniaxial stretching (figs. S27 and S28), making them also suitable for engineering specific mechanical properties. Notably, these mechanical properties can be further customized by extending the current topology optimization framework to multi-objective optimization tasks, which could enable the simultaneous optimization of both geometry and mechanical performance, as detailed in text S5 and fig. S29. In addition, a fully auxetic behavior of the multilayer 2D network with both in-plane and out-of-plane negative Poisson’s ratios can be potentially achieved by optimizing the 2D precursor pattern, as discussed in text S6 and figs. S30 and S31.

### Demonstrative application in reconfigurable volumetric 3D display

**Fig. 6.**
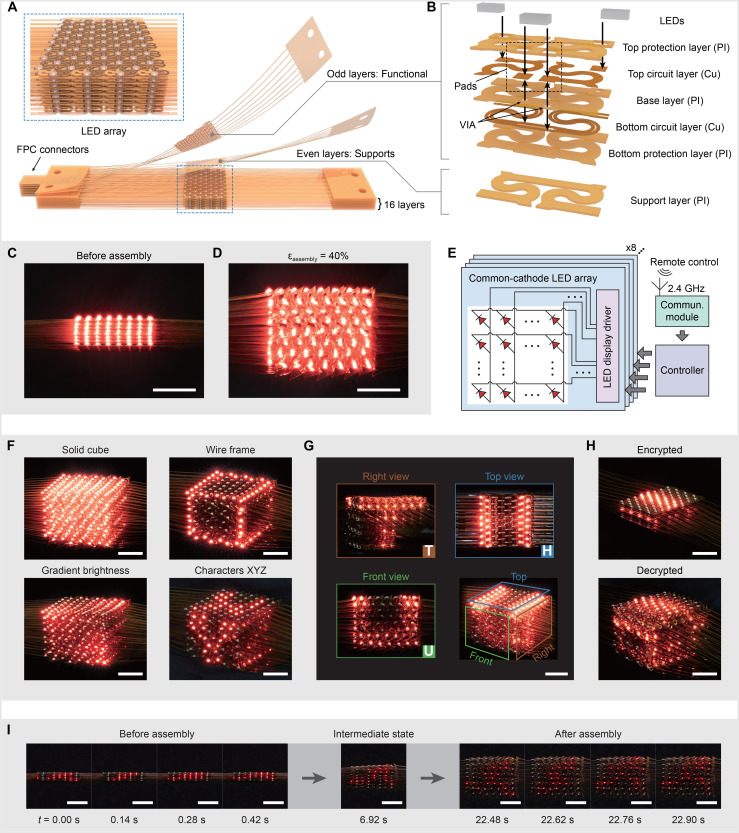
Demonstrative application in reconfigurable volumetric 3D display. (**A**) Schematic diagram of the stacked 2D network precursors integrated with LED arrays. (**B**) Expanded view of a unit cell of the device. (**C** and **D**) Volumetric 3D display before (C) and after (D) assembly with 40% strain. (**E**) Block diagram of the LED array system, including the driving and control modules. (**F**) Four representative 3D patterns displayed by the device, including a solid cube (top left), a cube wire frame (top right), a cube with gradient brightness (bottom left), and three characters “X,” “Y,” and “Z” on different faces of the cube (bottom right). (**G**) Demonstration of displaying a 3D pattern, which appears as different characters when viewed from different perspectives. (**H**) Demonstration of information decryption using the reconfigurable volumetric 3D display, where the two images correspond to the states before and after assembly with 40% strain. (**I**) Image sequence of the reconfigurable volumetric 3D display playing an animation of an athlete running rightward during the assembly up to 40% strain. Scale bars, 1 cm [(C) to (H)] and 2 cm (I). VIA, vertical interconnect access; FPC, flexible printed circuit.

The compatibility of the proposed tensile buckling strategy with planar microfabrication technologies allows integration of inorganic electronic devices with 3D network materials for various applications. Figure 6 (A and B) provides expanded views of the design illustration for a reconfigurable volumetric 3D display. This device consists of 16 layers, in which the functional layer and the support layer are stacked in an alternative manner (see fig. S32 for details). Their overall structural patterns are the same as the optimal S-shaped patterns in Fig. 4I. The functional layer has a laminated construction of bottom protection layer (PI, 25 μm), bottom circuit layer (Cu, 12 μm), base layer (PI, 25 μm), top circuit layer (Cu, 12 μm), top protection layer (PI, 25 μm), and commercial light-emitting diodes (LEDs) (0.35 mm in thickness and 1 mm by 0.5 mm in lateral size) (Fig. 6B). Here, the ribbon width is 500 μm for all three PI layers, and the widths of metal wires are 150 μm for bottom circuit and 350 μm for top circuit, respectively. Through tensile assembly (ε_assembly_ = 40%), the 16-layer device precursor is transformed into a 3D network architecture (Fig. 6, C and D), such that the 512 LEDs are distributed quite uniformly in the 3D space, with an average spacing of 3.5 mm along all three orthotropic directions (*x*, *y*, and *z* axes). When integrated with the control module (Fig. 6E and figs. S33 and S34), the 3D architected device can display dynamically a diversity of 3D patterns (Fig. 6F and movie S4).

Conventional technologies of glasses-free 3D display—such as holography (*70*, *71*), swept-volume display (*72*, *73*), and techniques relying on controlled particles (*74*, *75*)—have achieved rapid developments recently, and some of them have been commercialized. However, these technologies also have certain limitations, such as limited ranges of perspective and refresh rate (table S2). In comparison, the developed volumetric 3D display can be viewed from any spatial angles, with the capabilities to display a 3D object with a high refresh rate (50 Hz). Figure 6G demonstrates a 3D pattern that can be displayed as “T,” “H,” and “U,” when viewed from *x*, *z*, and *y* directions. In addition, the volumetric 3D display is mechanically reconfigurable (*76*), owing to the reversible assembly process through repetitive tension and unloading. The reconfigurability allows the spatial resolution to be adjusted for information encryption/decryption. Figure 6H provides an example, in which the displayed information can hardly be discerned before the 3D assembly due to the small *z*-directional resolution (1 mm). Uniaxial tension (40%) enlarges this *z*-directional resolution to ~3.5 mm, allowing the displayed cabin to be discerned. In particular, the ratio between the *z*-directional resolution of the device in the stretched state and the stress-free state is a crucial factor for its reconfigurability. Figure 6I, fig. S35., and movie S5 demonstrate another example using a display device with an increased height ratio (doubled in comparison to that shown in Fig. 6, C and D). In this example, an athlete running rightward is displayed, which is difficult to identify in the stress-free state of the device but becomes recognizable when 40% tension is applied.

The volumetric 3D display can be also used in a range of other applications (fig. S36 and movie S4), such as a 3D spectrogram that can display the power spectral density of a music in a time dynamic manner and a 3D snake game. Furthermore, a range of different 3D temperature distributions can be created by leveraging the thermal effect of the 3D LED array (fig. S37).

## DISCUSSION

In summary, the unique tensile buckling strategy and the data-driven topology optimization method allow access to 3D network materials with optimal ribbon architectures that yield maximized interlayer separations. The resulting 3D network materials offer highly tunable J-shaped stress-strain curves, in which the anisotropy can be also adjusted by rationally tailoring the design parameters. The excellent compatibility of the tensile buckling strategy with mature planar microfabrication techniques allows a diversity of high-performance materials to be incorporated into the 3D network for different uses. The demonstrated reconfigurable volumetric 3D display suggests promising potential for applications in 3D display, biointegrated electronics and tissue engineering. For instance, further studies could follow by using micropatterned biodegradable materials and electronic components to construct 3D electronic scaffolds that can enhance soft tissue regeneration by reducing the mechanical mismatch between scaffold and tissue while also offering monitoring/stimulation capabilities. In addition, the overall geometry of the assembled 3D network structure can be engineered through the gradient control of precursor design, with an example of teardrop-shaped 3D network structure demonstrated in text S7 and figs. S38 and S39. Inverse design methods can be further developed to achieve specific height distributions within the entire network structure. While the developed 3D network materials offer excellent mechanical properties along *x* and *y* directions, they show low mechanical strengths under uniaxial stretching along the out-of-plane direction. Such a weakness can be addressed by adding elastomer encapsulation to the 3D network (fig. S40) or introducing covalent bonding selectively between adjacent layers.

## MATERIALS AND METHODS

### Fabrication of the 3D network material

Preparation of the 3D network material began with fabrication of each planar precursor layer (fig. S41). The process involves spinning coating of a thin poly(methyl methacrylate) (PMMA) layer (400 nm) and then a PI layer (5 to 25 μm). Photolithography and lift-off techniques defined patterned mask (Ni), which allowed reactive-ion etching of PI. Dissolving the sacrificial layer of PMMA released the patterned PI layer from the Si wafer, which completed the preparation of the patterned PI layer. Repeating the above process allowed preparation of the desired number of PI layers. Afterward, adding an adhesive at two ending pads of a PI layer through doctor blade, followed by transfer printing of another PI layer using a water soluble tape [polyvinyl alcohol (PVA)], bonded two PI layers together. Repeating this process enabled fabrication of multilayer patterned PI precursor. Uniaxial stretching of the multilayer patterned PI precursor using a customized mechanical stage while controlling the strain amplitude transformed the multilayer precursor into 3D network configuration. After placing the mechanical stage into an oven, heating the network material to 220°C, followed by cooling to room temperature, completed the fabrication of freestanding 3D network material.

### FEAs of the 3D assembly process

FEAs based on commercial software (ABAQUS) were exploited to model the process of 3D assembly through the tensile buckling. Shell elements (S4R) were adopted for the multilayer precursor structure, with refined meshes to ensure the convergence of simulations. Explicit solver was used to take into account the complex interlayer contact behavior during the nonlinear deformation process. A hard contact with a frictional coefficient of 0.1 was used in the simulations, noting that the variation of frictional coefficient in the range of [0.05, 0.2] does not lead to any notable change in the buckling deformations or stress-strain curves (text S8 and fig. S42). Appropriate mass scaling enabled improved computational efficiency of explicit solving using FEA. Displacement-controlled uniaxial loadings were exploited, consistent with the loadings applied in experiments, such that same levels of uniaxial stretching were applied to all layers of planar precursors. Spring/dashpots were introduced to connect adjacent precursor layers at the loading ends, where a proper spring stiffness was adopted to ensure sufficient interlayer contact while avoiding disturbance on the structural deformations (fig. S43). The dashpots can effectively prevent the occurrence of evident snap through during the buckling deformations.

### Data-driven topology optimization

Data-driven topology optimization relied on FEA to solve the forward problem, and exploited the differential evolution algorithm to search for the optimal solution. A mature library (scipy.optimize.differential_evolution) of differential evolution algorithm was exploited, which can support multiple workers for parallel computations. The parameter setting for the differential evolution algorithm is detailed in text S9. The ABAQUS CAE script allowed automatic modeling, computation, and data extraction. The architecture of topology optimization program is detailed in fig. S44 and text S10.

### Characterization of mechanical properties

The uniaxial stress-strain responses were measured using a dynamic mechanical analyzer (DMA, TA Instrument, US). A low strain rate (~0.2%/s) ensured that the viscoelastic effect of the material (e.g., PI and PET) can be neglected. Transverse strains (along *y* and *z* directions) induced by the *x*-directional stretching was determined on the basis of the analyses of photos taken during the stretching.

### Reconfigurable volumetric 3D display

Each layer of planar precursors of the device was prepared using the techniques of flexible printed circuit board (fig. S32). The control module (figs. S33 and S34) consists of a microcontroller unit (MCU, STM32F030), eight drivers of LED array, and a bluetooth module. The LED array on each of the eight layers is independently controlled by an LED array driver. The driver operates by illuminating the LEDs in a row-by-row scanning manner (with a refresh rate of 800 Hz) to display the desired pattern. These eight LED array drivers are all controlled by the MCU, which receives signals from the Bluetooth module and subsequently transmits them to the registers of the LED drivers. Through remote computer control, the device can dynamically display 3D objects.

## Supplementary Materials

### The PDF file includes:

Texts S1 to S10 Figs. S1 to S44 Tables S1 and S2 Legends for movies S1 to S5 References
