# Supplementary material for: Rational assembly of 3D network materials and electronics through tensile buckling
Source: Sci Adv. 2025 Sep 10;11(37):eadz0718. doi: 10.1126/sciadv.adz0718 (PMC12422206; doi:10.1126/sciadv.adz0718)
Supplement: Supplementary file 1 — Texts S1 to S10 Figs. S1 to S44 Tables S1 and S2 Legends for movies S1 to S5 References [file sciadv.adz0718_sm.pdf]

Science Advances
AAAS

Supplementary Materials for

Rational assembly of 3D network materials and electronics through tensile buckling

Xiaonan Hu *et al.*

Corresponding author: Yihui Zhang, yihuizhang@tsinghua.edu.cn

Sci. Adv. **11**, eadz0718 (2025)
DOI: 10.1126/sciadv.adz0718

The PDF file includes:

Texts S1 to S10
Figs. S1 to S44
Tables S1 and S2
Legends for movies S1 to S5
References

Other Supplementary Material for this manuscript includes the following:

Movies S1 to S5

Supplementary Text

Supplementary Text S1. Assembly of 3D network materials using two different boundary constraints

Fig. S2 presents FEA results of tensile assembly using two different types of boundary constraints: (1) connecting the network sample directly to the loading pads at the left and right edges, and (2) introducing a set of long parallel ribbons (e.g., with length of $L_{\text{extension}} = 2L_0$) between the loading pads and the network sample. The first boundary constraint leads to a pillow-shaped 3D structure with pronounced boundary effects, while the second boundary constraint results in a more uniform, cuboid-like 3D geometry with better periodicity. Notably, the total heights of the network structures in both cases are very similar, indicating that the out-of-plane deformation in regions away from the boundaries is largely unaffected by the boundary constraint.

For the network structures shown in Figs. 1, 2, and 5, the first boundary constraint is used to demonstrate the concept of the 3D assembly and to characterize the mechanical properties of the freestanding network material, where a perfect periodicity is not essential. Despite the boundary effects, the periodicity of the interior regions is not significantly affected, allowing for reliable investigation of the geometry and mechanical properties of the network materials. Furthermore, introducing additional parallel straight ribbons would increase the overall dimension of the entire 2D precursor. Differently, for the 3D display device shown in Fig. 6, the second boundary constraint is used to create a 3D network with a better periodicity, which is important for the practical application.

Supplementary Text S2. Influence of interlayer compression on the assembly of multilayer network structure

The interlayer compression arising from geometric and boundary constraints play a crucial role in the assembly of multilayer network structures. According to our observations of the deformation process, the interlayer compression has two main effects: (1) directly influences the deformed configuration of unit cells in each layer, and (2) affects the vertical alignment of unit cells across different layers.

Near the left and right boundaries, the compression is substantial due to mechanical constraints of loading pads. As a result, unit cells close to the boundaries exhibit limited out-of-plane deformation compared to those in the middle region. This restriction leads to a pillow-like shape, where the height of unit cells near the boundary is limited. In contrast, in the middle region, which is far from the boundaries, the interlayer compression is much smaller. This is evidenced by the relatively uniform height observed in the middle region (fig. S5). Here, the out-of-plane deformation of the unit cells is largely unaffected by interlayer compression, resulting in similar deformations of the inner and outermost layers.

The interlayer compression can also induce slippage, leading to the vertical misalignment of unit cells. Simulations of a 16-layer structure (fig. S3B) show a pronounced pillow-like shape, with a very narrow middle region of uniform height. The normalized total height of the 16-layer structure is 8.1, which is much lower than twice that of the 8-layer structure (2×6.1), as a severe misalignment can be observed. The outermost layers are slightly more stretched due to the convex shape, which further reduces the structural periodicity of the 3D network material along the out-of-plane direction.

Supplementary Text S3. Thickness effect on the maximum out-of-plane deformation of the structure

Based on the optimized ribbon-based precursor design, we conducted a series of simulations on precursors with the same patterns but different normalized thickness, as shown in fig. S9. The results show that the maximum normalized heights achieved by structures with normalized thickness of 0.025, 0.05, and 0.1 are very close to each other, indicating that increasing the thickness of the 2D precursor generally has a negligible effect on the assembled 3D geometry. However, the thickness has a significant effect on the maximum stress in the structure. Specifically, when the assembly strain is relatively small and the deformation is in the bending- and torsion-dominated stage, the maximum stress increases linearly with increasing the thickness under the same assembly strain. As the assembly strain increases, the deformation becomes tensile-dominated, and the maximum stress becomes largely independent of thickness under the same assembly strain. The increase in thickness reduces the stretchability of the structure, and can limit its out-of-plane deformation capability before failure.

Therefore, since the optimization aims to maximize the out-of-plane deformation before failure, the thickness only becomes important if it is too large and causes early failure. As long as the normalized thickness is within a reasonable range (e.g., below 0.1), it has negligible effects on the optimization results.

Supplementary Text S4. Precursor design based on the results of topology optimization

The initial assumption for the 2D periodic precursor is an array of straight and curved ribbons, as shown in Fig. 3A. The curved ribbons serve as the functional structure to deform out-of-plane when stretched along the *x*-axis direction, while the vertical straight ribbons serve as connectors and contribute very slightly to the out-of-plane deformation. The array is designed to be symmetric along both *x*- and *y*-axes (this type of symmetry belongs to the wallpaper group 'pmm'). These symmetries simplify the analysis of the unit cells by requiring zero rotation of at the boundary, which is easier to handle than general periodic boundary conditions.

Based on this design, a curved ribbon is considered in the topology optimization in Fig. 3. The optimization result shows a S-shaped feature (Fig. 3F). This feature remains consistent even when changing the parametrization to kirigami-type or levelset-based scheme (figs. S11 to S14), indicating its importance for achieving significant out-of-plane deformation. This is mainly because the middle section of its zigzag pattern rotates under stretching, resulting in notable height, and the smooth design avoids sharp turns, reducing stress concentration, allowing maximum height to be achieved before failure. Consequently, the S-shaped feature is crucial for achieving a large out-of-plane size through the tensile 3D assembly.

When there are many layers of precursors, especially when $n > 6$, the interlayer compression (which arises from boundary effects) can affect the assembled configuration. If the deformed 3D structures lack resistance to the out-of-plane pressure, the height of the assembled network material cannot be maintained. Therefore, the curved ribbon structures should provide sufficient compressive rigidity in the height direction, which is, however, very difficult to consider in the topology optimization. This compressive rigidity is mainly determined by the ribbon width and the areal density of S-shaped ribbons in the entire planar network. As such, directly placement of the S-shaped ribbon into the periodic network shown in Fig. 3A is not optimal, as the vertical

spacing between S-shaped ribbons is large, leading to a reduced areal density of S-shaped ribbons. A solution to increase the areal density S-shaped ribbons is to eliminate the vertical straight ribbons and directly join the S-shaped ribbons in the y-axis direction. By reducing the array distance in the y-axis direction, the S-shaped ribbons can be directly joined together by sharing their horizontal segments at the beginning and ending, resulting in the final periodic network design shown in Fig. 3G. It is also noteworthy that the final design belongs to the wallpaper group 'cmm'.

The results of topology optimization with maximized interlayer separation (Fig. 4) show a similar S-shaped feature as those in Fig. 3. Additionally, the odd and even layers exhibit mirror symmetry, which is also observed in the topology optimization based on the levelset-based scheme (figs. S16 and S17) and topology optimization with an alternative objective function (fig. S18). The mirror symmetric S-shaped pair ensures a large interlayer separation by increasing the topology difference between patterns in adjacent layers, thus breaking the similarity in deformations of adjacent layers. By contrast, identical patterns in all different layers cause synchronous 3D deformations, resulting in tightly stacked layers that fail to form network materials with notable height (Fig. 4I and fig. S19).

Supplementary Text S5. Multi-objective optimization for both geometry and mechanical performances

To demonstrate the capability of our topology optimization framework for simultaneous engineering of both shape and mechanical properties, we performed a multi-objective optimization on a single-layer structure with a levelset-based pattern design. The optimization targeted three main objectives (fig. S29A): (1) achieving a stretchability not lower than 40%, (2) maximizing the normalized height during assembly, and (3) minimizing the deviation (measured by root mean square error, RMSE) between the normalized force-assembly strain curve and a user-defined quadratic target curve.

An objective function was constructed to account for these three targets (as illustrated in fig. S29A), including a penalty factor for stretchability below the threshold and weight factors to balance the height and force-displacement objectives.

The optimization converged within 600 iterations (fig. S29B) and produced promising results (fig. S29 (C and D)). The optimized structure achieved a stretchability of 43% (fig. S29E), and reached a large normalized height of 1.209, which is only slightly smaller than the result of pure height optimization ($(h / l_0)_{max} = 1.320$, fig. S14). The normalized force versus assembly strain curve closely matched the target curve. These results demonstrate the effectiveness of our multi-objective optimization approach in simultaneously engineering both the shape and mechanical properties of the network structure.

Supplementary Text S6. Auxetic behavior with both in-plane and out-of-plane negative Poisson's ratios

Auxetic behavior can offer unique advantages for applications in deployable solar cells, biomedical electronics and other areas. A fully auxetic behavior requires the architected material to offer negative Poisson's ratios in two orthogonal directions. Based on the proposed tensile buckling strategy, we have conducted two sets of optimizations to explore the possibility of achieving both in-plane and out-of-plane negative Poisson's ratios, as detailed below.

The first set of optimization focused on minimizing the in-plane Poisson's ratio of a single-layer structure with a ribbon-based pattern design. In this task, the objective function is defined as the average in-plane Poisson's ratio prior to failure (fig. S30A), aiming to achieve its minimization. Here, the average in-plane Poisson's ratio is defined as the slope of linear fitting for the curve of $\Delta y_{pp} / y_{pp0} - \varepsilon_{assembly}$ in the assembly strain range of [0, min{ε_{fail}, ε_{ub}}], where Δy_{pp}, y_{pp0}, ε_{fail}, and ε_{ub} are illustrated in fig. S30A. The structure was subjected to assembly strains of up to 50%, as higher strains would lead to a stretching-dominated regime that tends to induce a positive Poisson's ratio. The optimization converged after 3650 iterations, with representative intermediate results shown in fig. S30B. It can be observed that structures achieving significant negative in-plane Poisson's ratios generally have very narrow widths, leading to nearly pure in-plane deformations during stretching (fig. S30C). These structures show a large negative Poisson's ratios at the beginning of stretching, but fail shortly after, exhibiting very low stretchability (fig. S30D). Additionally, these structures show negligible out-of-plane deformations during stretching. These findings indicate that pronounced negative in-plane Poisson's ratios are primarily associated with in-plane deformation mechanisms.

In the second set of optimization (fig. S31), the objective remains as the average in-plane Poisson's ratio, but we introduce an additional penalty to ensure that the level of out-of-plane deformation satisfies a minimum requirement ($(h / l_0)_{max} \geqslant 0.5$) (fig. S31A). This optimization produces an optimal structure with a much lower negative Poisson's ratio (–4.6) compared to the result from the optimization focusing solely on negative Poisson's ratio (–16.5) (fig. S30B). Nevertheless, this optimal structure exhibits both in-plane and out-of-plane auxetic behavior. As shown in Fig. R11 (C and D), when the tensile strain is 7%, the deformed structure exhibits a greater span along both *y*- and *z*-axis compared to its initial configuration, as evidenced by the values of $\Delta y_{pp} / y_{pp0} = 1.32$ and $h / l_0 = 0.23$. In particular, the in-plane auxetic behavior is attributed to in-plane bending before out-of-plane buckling. Once the out-of-plane buckling occurs, further increase of the *y*-axial span stops as the deformation mode transitions from in-plane bending to out-of-plane buckling. For instance, as the tensile strain reaches 7.9%, the ratio $\Delta y_{pp} / y_{pp0}$ decreases to 1.15, and h / l_0 increases to 0.50. These results highlight the possibility of designing structures that exhibit both in-plane and out-of-plane auxetic behavior.

Supplementary Text S7. Influence of precursor geometry on material height and strategies to engineer the height distribution

By tailoring the geometric parameters (w / l_0, t / l_0 and l_c / l_0) of the 2D precursor presented in Fig. 3G, the mechanically properties of the assembled network material can be finely tuned. Notably, the anisotropy of the network material is mainly governed by the normalized effective length (l_c / l_0). However, this geometric parameter is also closely related to the normalized height of the network material, necessitating careful consideration of the balance between height and anisotropic mechanical behavior.

Fig. S38 shows the relation between the normalized total height (H_m / l_0) of eight-layer precursors and the normalized effective length (l_c / l_0). A short effective length reduces the total height of the network material, and narrows the range of deterministic stage. This is because the interlayer separation is contributed mainly by the deformed central segments of the S-shaped microstructures. A shorter effective length results in shorter rotated segments, thereby reducing the interlayer separation.

Other geometric parameters exhibit very minor influences on the height during the 3D assembly, allowing the mechanical properties to be tuned independently, without affecting the 3D configuration of the network material. For instance, the elastic modulus and modulus ratio are mainly governed by the normalized width (w / l_0) and thickness (t / l_0) (Fig. 5 (B and C)) which determine the tensile and bending stiffness of the curved ribbon. These two parameters have negligible influences on the 3D geometry and overall size of the network material.

By varying the distribution of the effective length in the precursor design, the height profile of the assembled network structure can be precisely controlled. For example, using precursors with gradient pattern designs allows for the assembly of teardrop-shaped network structures (fig. S39). This strategy enables tailored height distributions within the network, offering greater flexibility in customizing the 3D geometry to meet specific application requirements.

Supplementary Text S8. Influence of friction on the assembly process and mechanical properties of network materials

FEA were conducted to quantitatively assess the influence of friction on the assembly process and mechanical properties of network materials. Fig. S42A compares the normalized total height during tensile assembly of multilayer precursors with a set of different frictional coefficients. When the assembly strain is below 40%, there are negligible differences in the normalized total height of the network material, suggesting that the deformation of the multilayer precursors occurs in a highly deterministic manner, where the friction play very minor influences. This highlights the precision in controlling the height of the network material during the deterministic stage.

When the assembly strain exceeds 40%, notable differences in the total heights of the network material assembled from precursors with different frictional coefficients can be observed. Specifically, in the frictionless case ($\mu = 0$), there is an abrupt drop in the height curve, indicating a relatively synchronized collapse of all interlayer contacts. In contrast, in cases of a finite friction (e.g., 0.05 to 0.2), the total height decreases progressively as the assembly strain increases, suggesting that the collapse occurs in a random and gradual manner. This comparison highlights the distinction between frictionless and frictional conditions. However, when the frictional coefficient varies from 0.05 to 0.2, there is no significant differences in the total height, and the height variations are primarily attributed to random collapse behavior.

The assembly strain-height curves from FEA results of multilayer precursors with consideration of frictions agree well with the experimental results (Fig. 1E), validating the accuracy of the FEA.

In this work, both PI (Fig. 1B) and PET (fig. S6) are utilized as the composing materials for the preparation of 3D network materials. As the material parameter has a negligible effect on the resulting 3D configuration, their network configurations are basically unaffected by the choice of PI or PET, given that their frictional coefficients are both below 0.2.

Fig. S42B shows the FEA results of the force response during assembly of the network material based on precursors with different frictional coefficients. Except for the local imperfection in the force curve of the frictionless case, there is no significant difference between the force curves for different frictional coefficients.

The consistency in force responses can be explained by the mechanism through which the friction influences the deformation process of the network materials. The friction introduces enhanced local randomness to the microstructure deformation, disrupting the synchronized collapse of interlayer contacts and smoothing the height response. However, the force during

stretching primarily depends on the microstructure deformation rather than the interaction between adjacent layers, resulting in similar force responses across all cases.

The local imperfection in the force curve in the frictionless case can also be attributed to the lack of randomness, which leads to abrupt changes in the network topology, resulting a more noticeable local imperfection. In contrast, the curves in the frictional cases are smoother.

This insensitivity of the force response to the friction forms the basis for analyzing the mechanical properties of the as-fabricated network material presented in Fig. 5.

Supplementary Text S9. Parameter setting for the differential evolution algorithm

A widely used differential evolution strategy ('best1bin') is exploited, with the mutation rate specified as dithering within the range of [0.5, 1.0] and the crossover probability specified as 0.7.

The population size is specified as 15 times larger than the number of optimization parameters (e.g., if there are 20 optimization parameters, there are 300 individuals in each generation). The end condition of the optimization is met when the relative standard error of a generation is less than 2%. The relative standard error of a generation is calculated as STD(y_i) / ABS(MEAN(y_i)), where y_i is the fitness of the i-th individual in the generation, STD(y_i) is the standard error of y_i, and ABS(MEAN(y_i)) is the absolute mean value of y_i. The 2% tolerance is adopted, because once the relative standard error falls below 2%, the geometric features of precursor patterns remain almost unchanged in subsequent iterations.

The population initialization is performed by Hypercube sampling, maximizing coverage of the available parameter space. Since multiple workers are used, the best solution vector is updated once per generation, instead of being continuously updated within a single generation.

Supplementary Text S10. Architecture of the parallel optimization program

The optimization program is highly modular, as shown in fig. S44. The architecture consists of four main modules, namely, optimizer, load balancer, executor, and database.

The optimizer and load balancer are implemented using the differential evolution function from the open-source SciPy library, which enables parallelization with multiple workers, ensuring optimal performance and scalability.

The database used is SQLite, which ensures data integrity and provides reliable data persistence. SQLite can handle simple concurrent read/write operations, making it compatible with multiple workers. Despite its weak concurrency performance, it is sufficient for the optimization tasks in the current study, because the FEA computation is much slower than the database operations. The database contains two tables – one table stores the iteration process of differential evolution, which logs the best individual accumulated up to each iteration, and the other stores the fitness values of all ever-computed genes.

The executor is a user-defined function that encapsulates pattern generation, modeling, FEA, and post-processing, which are mainly implemented through automatically generated ABAQUS CAE scripts.

A caching mechanism is introduced to the executor. Once the fitness of an individual is calculated, it is stored in the database. If the individual is encountered again, its fitness will be directly retrieved from the database, instead of being re-calculated. Additionally, the caching mechanism enables restart of the optimization after interruption. By fixing the random seed of the

differential evolution algorithm, the entire iteration process can be fully reproduced once completed.

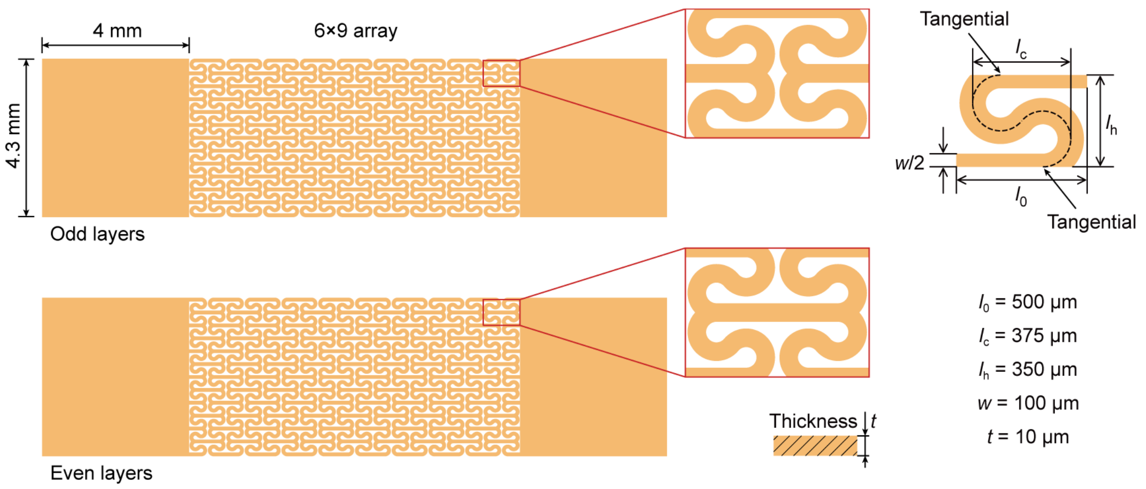

Fig. S1. Geometric parameters of the multilayer precursors presented in Fig. 1B.

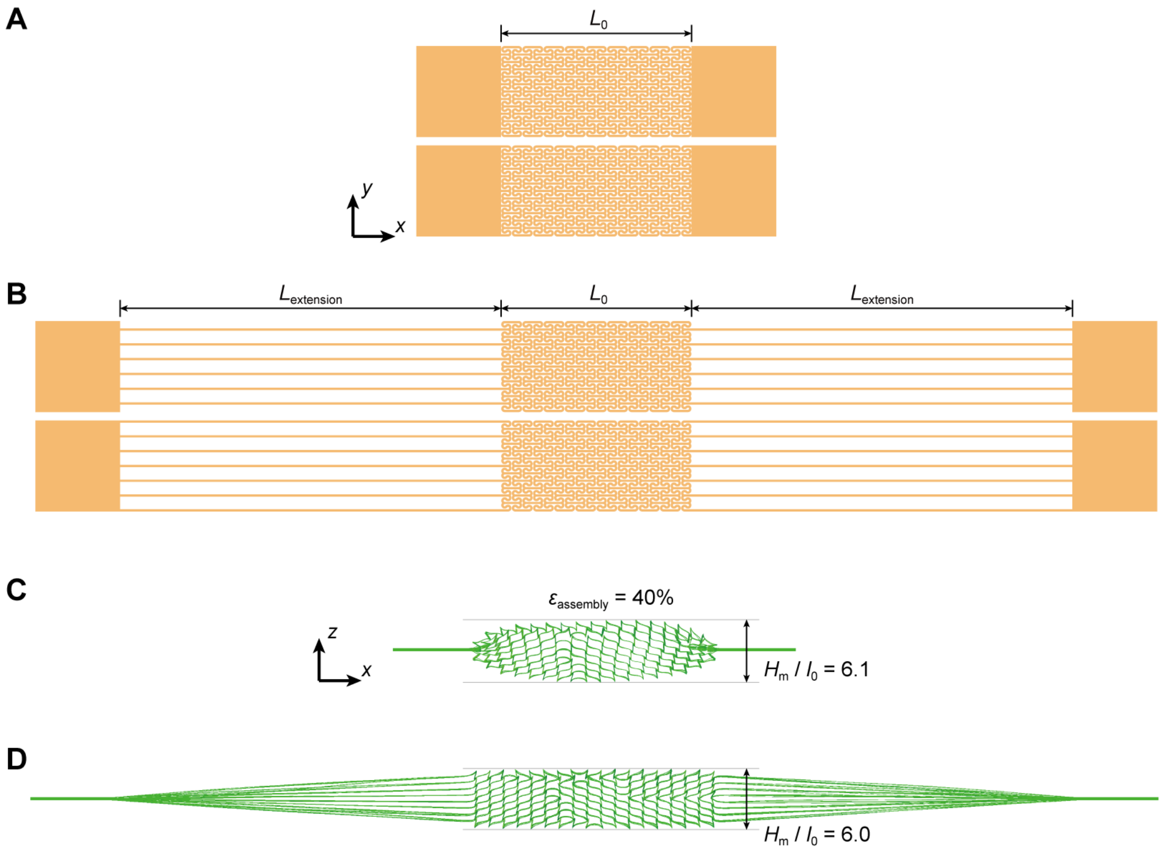

Fig. S2. Geometry comparison of network structures assembled from precursors with two different boundary constraints. (**A**) Precursor design with direct constraints at the left and right edges of the periodic pattern. (**B**) Precursor design with straight ribbons extended from the left and right edges of the periodic pattern. The x-axial lengths of the periodic pattern and the extended ribbons are annotated. (**C**) FEA result of an 8-layer network structure assembled from the precursor shown in (A). (**D**) FEA result of an 8-layer network structure assembled from the precursor shown in (B).

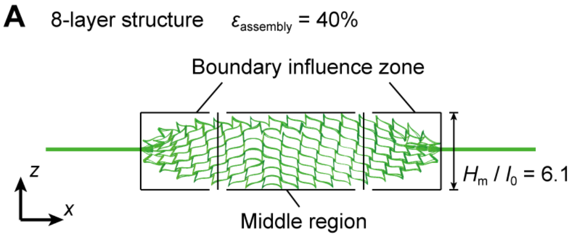

Fig. S3. Illustration of the boundary influence zone for an 8-layer structure (A) and a 16 layer-structure (B).

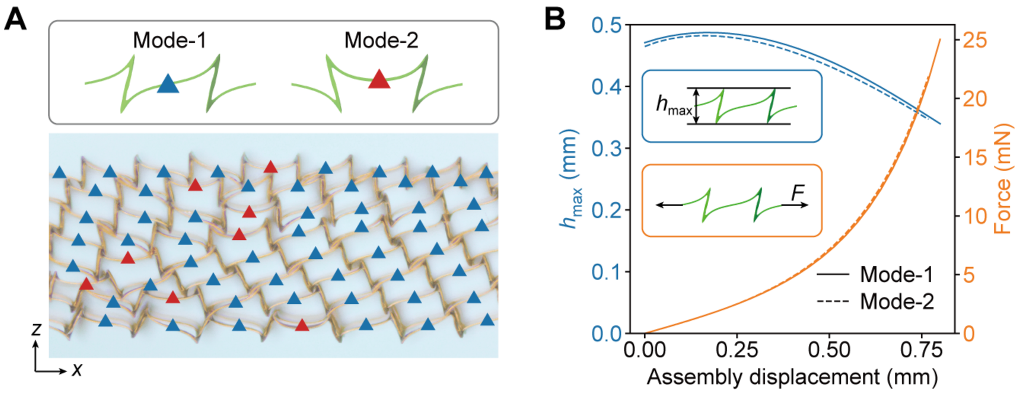

Fig. S4. Consistency of geometry and mechanical properties of different buckling modes. (**A**) Demonstration of two basic buckling modes, and their random occurrence in an assembled network. (**B**) Comparison between the geometric configuration and mechanical behavior of the two modes.

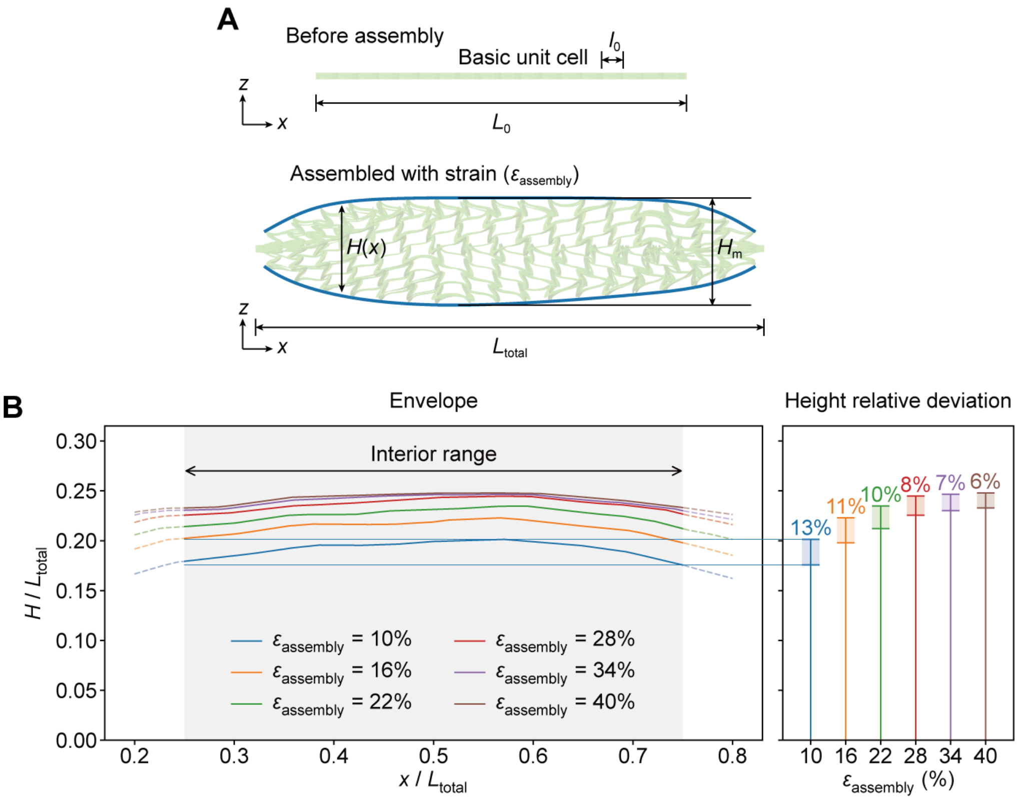

Fig. S5. Height uniformity of the assembled 3D material. (**A**) Definition of key geometric features of the network material before and after assembly. (**B**) Characterization of the height uniformity of the assembled network in *x*-axis direction. The left panel shows the envelope of the network and the right panel shows the relative height deviation of the structures with different level of assembly strain.

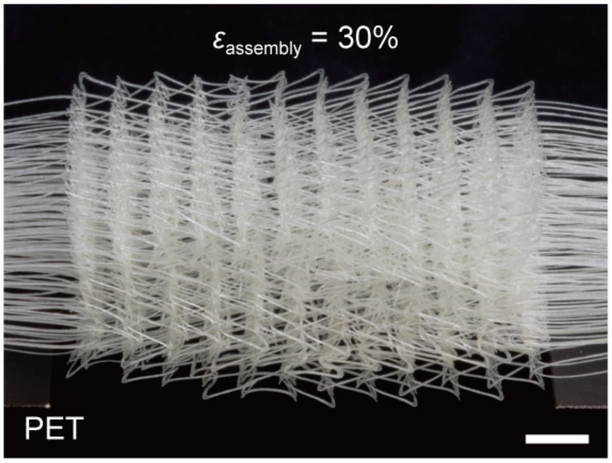

Fig. S6. 3D network material assembled based on PET material. The 3D network material is assembled from sixteen layers of the precursors shown in fig. S1 with 'AB' stacking. Scale bar, 5 mm.

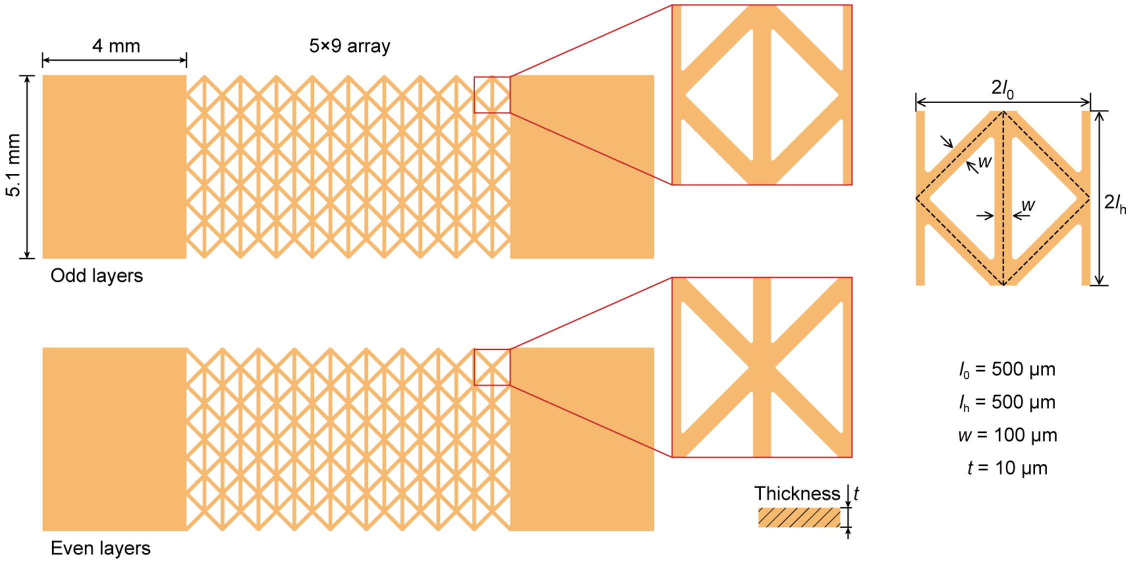

Fig. S7. Geometric parameters of the multilayer precursors presented in Fig. 2A.

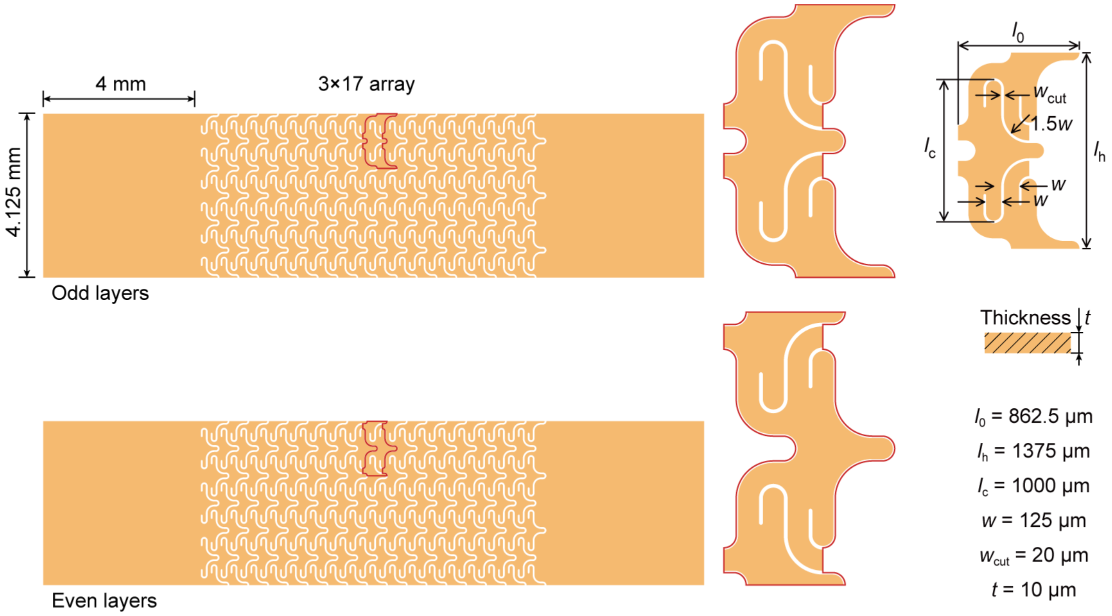

Fig. S8. Geometric parameters of the multilayer precursors presented in Fig. 2D.

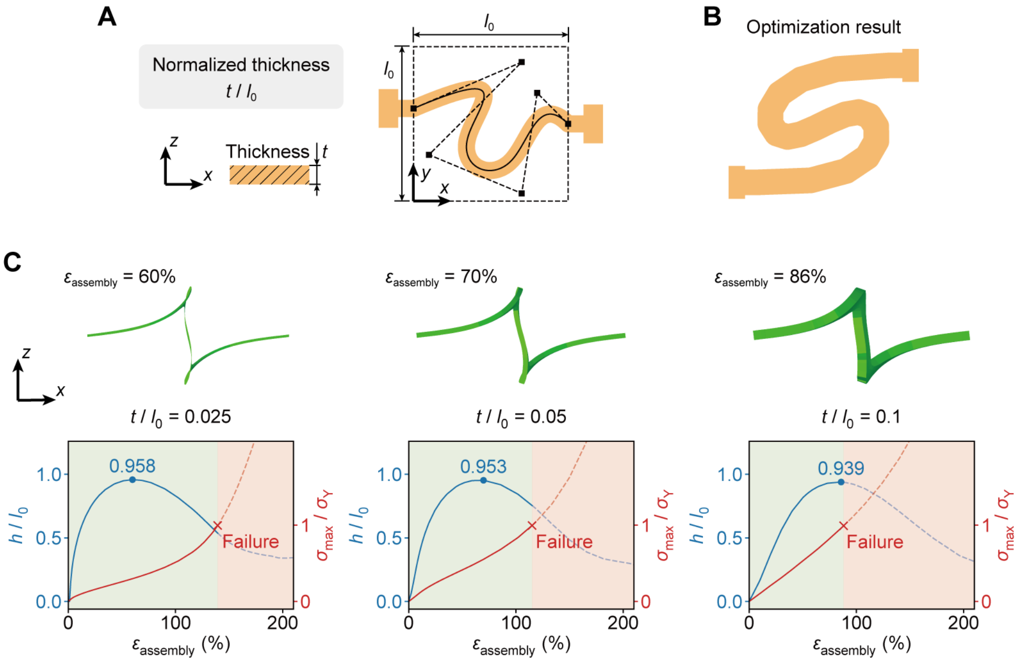

Fig. S9. Thickness effect on the maximum out-of-plane deformation of the structure. (**A**) Definition of the normalized thickness of the precursor. (**B**) Optimized ribbon-based precursor design, representing the pattern used for the thickness study in (C). (**C**) Deformed configurations of precursors with the same pattern but different normalized thicknesses at their corresponding maximum out-of-plane deformations, along with the relation of the normalized height and normalized Mises stress of the structure versus the assembly strain.

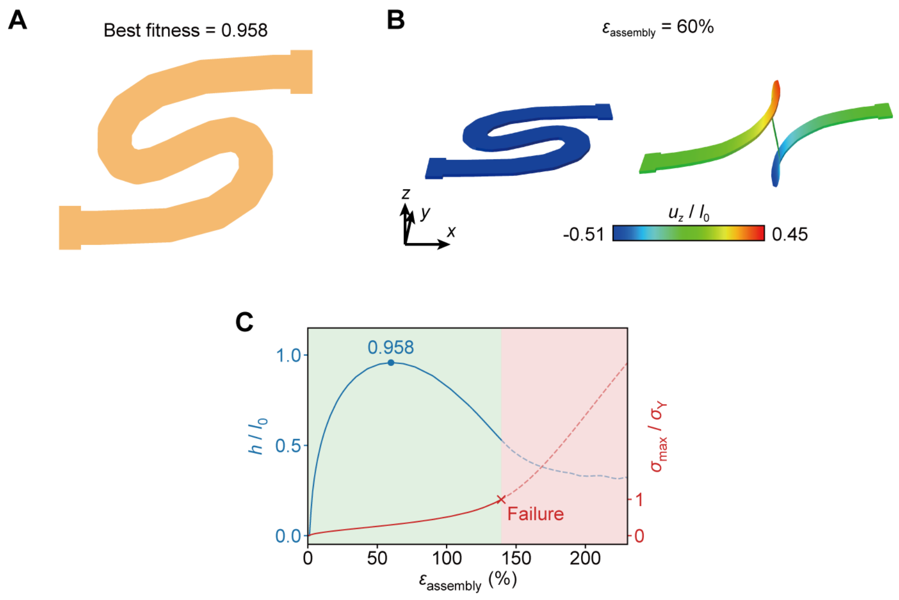

Fig. S10. Single-layer optimization results for the ribbon-based precursor designs. (**A**) Optimal pattern of the 2D precursor. (**B**) Deformed configurations of the optimal 2D precursor under the assembly strain that maximizes its height. (**C**) Relation of the normalized height and normalized Mises stress of the structure versus the assembly strain.

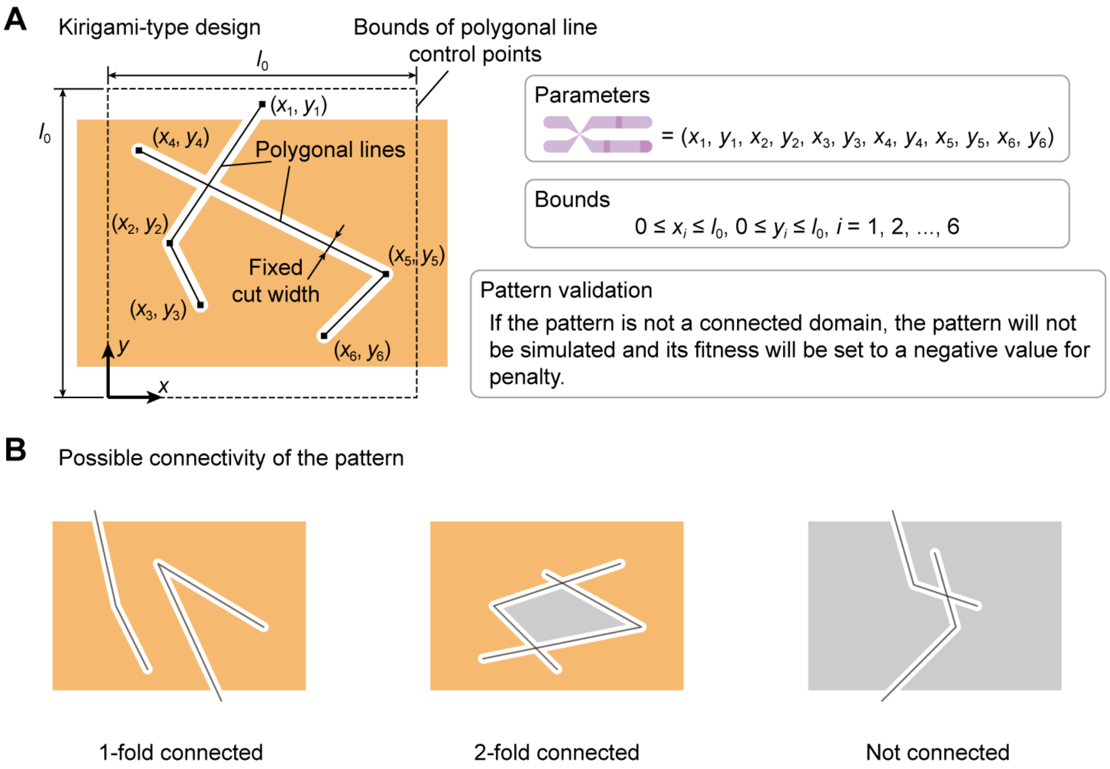

Fig. S11. Parametrization scheme for kirigami-type precursor patterns used for topology optimization. (**A**) Parametrization scheme for kirigami-type precursor patterns. (**B**) Possible connectivity of the kirigami-type pattern.

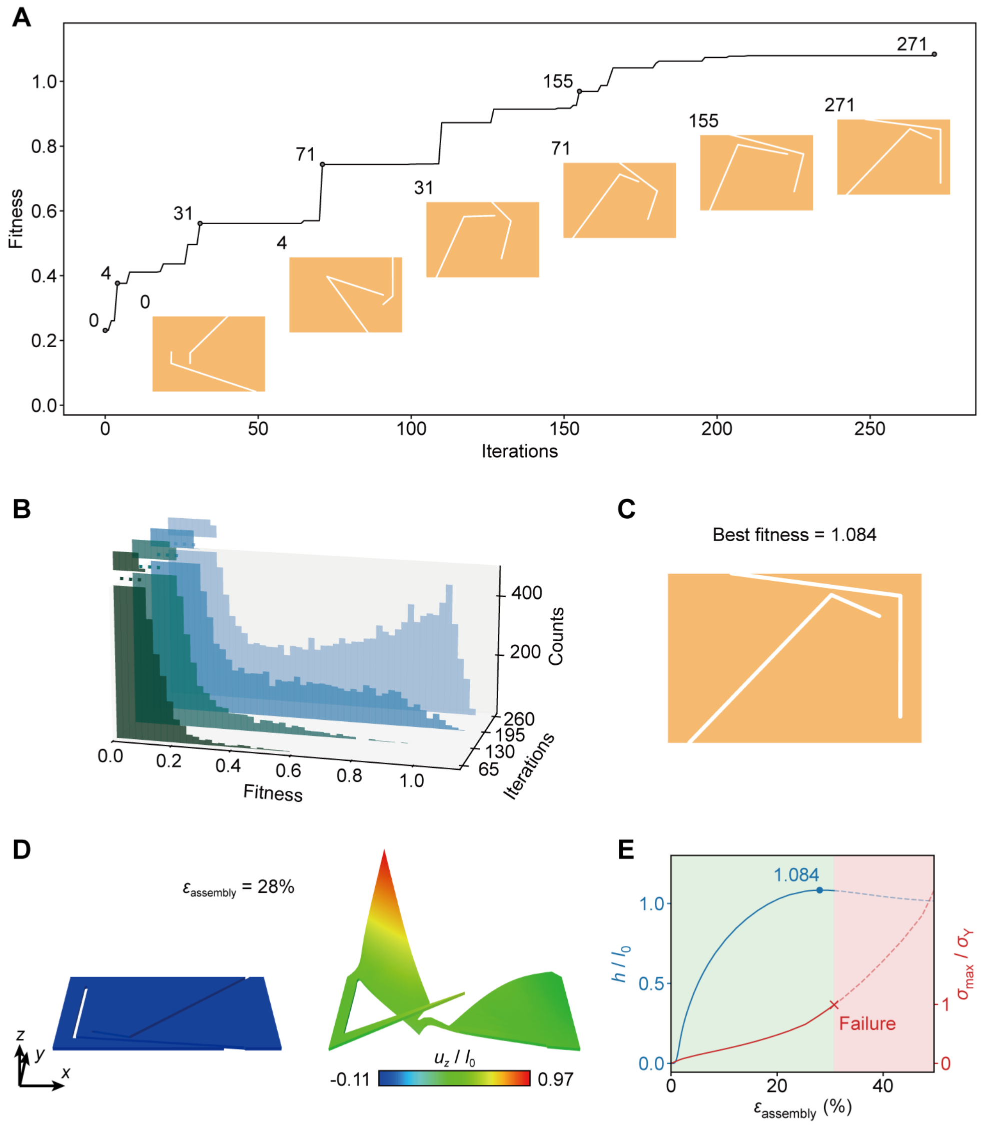

Fig. S12. Single-layer topology optimization of kirigami-type precursor designs. (**A**) Fitness evolution curve of the topology optimization, where some key intermediate results during iterations are marked and shown. (**B**) A set of histograms of the fitness of individuals accumulated to different stages of iteration. (**C**) Optimization result of the kirigami-type designs. (**D**) Deformed configurations of the optimal 2D precursor under the assembly strain that maximizes its height. (**E**) Relation of the normalized height and normalized Mises stress of the structure versus the assembly strain.

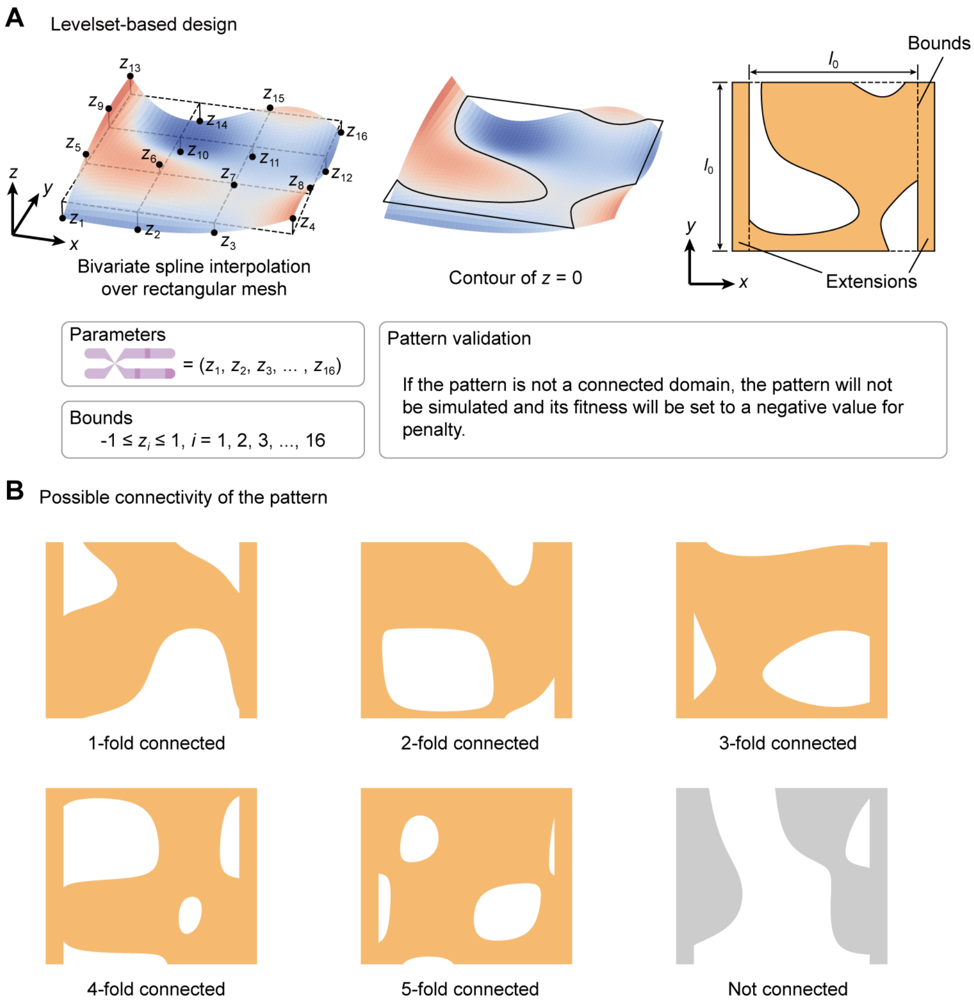

Fig. S13. Parametrization scheme for levelset-based precursor patterns used for topology optimization. (**A**) Concept of the levelset method for generating 2D regions to construct the 2D precursors, along with the parametrization of the levelset function. (**B**) Possible connectivity of the levelset-based pattern.

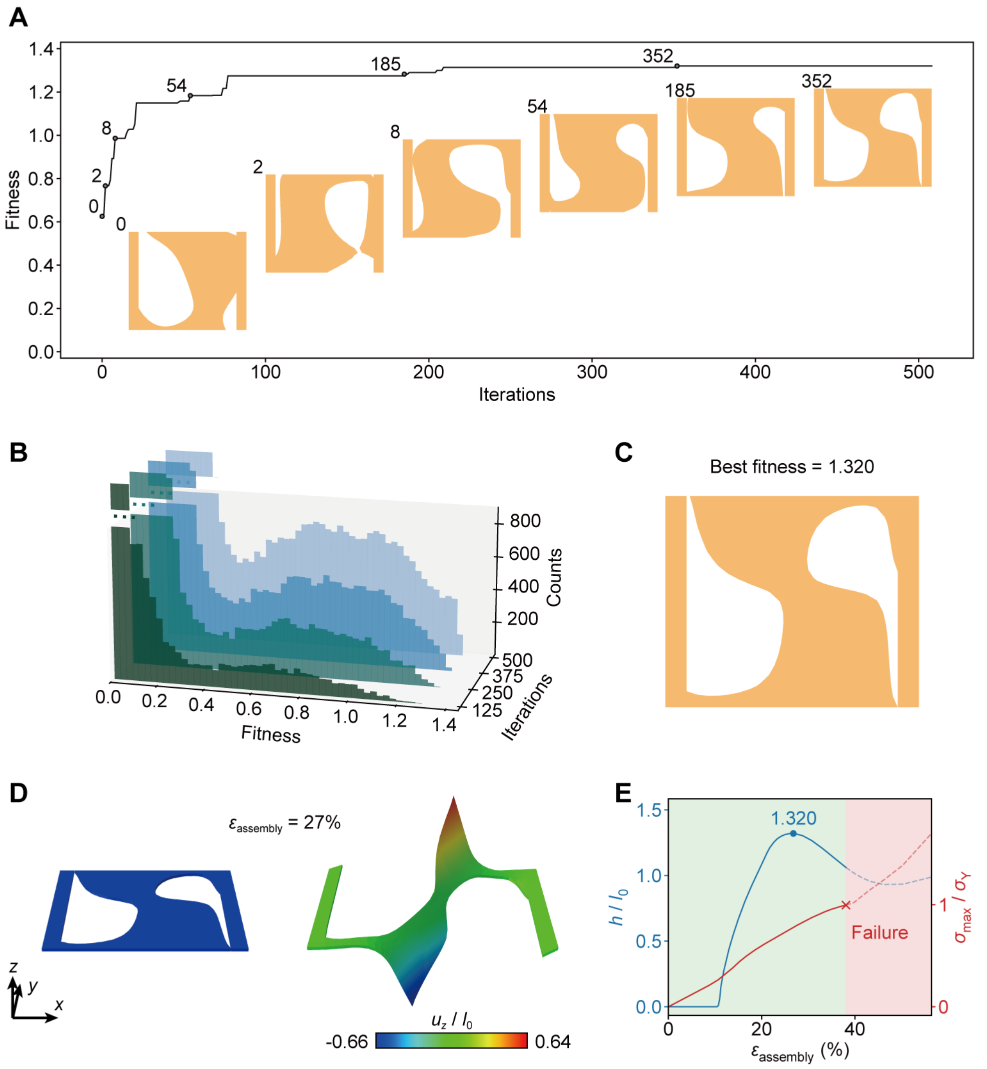

Fig. S14. Single-layer topology optimization of levelset-based precursor designs. (**A**) Fitness evolution curve of the topology optimization, where some key intermediate results during iterations are marked and shown. (**B**) Histogram of the fitness of all valid individuals. (**C**) Optimization result of levelset-based designs. (**D**) Deformed configurations of the optimal 2D precursor under the assembly strain that maximizes its height. (**E**) Relation of the normalized height and normalized Mises stress of the structure versus the assembly strain.

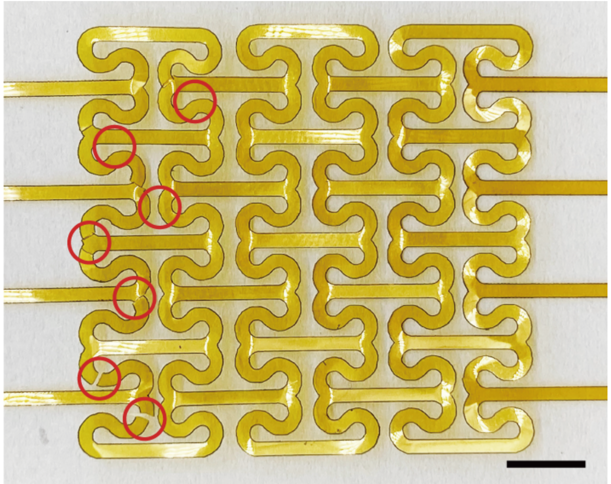

Fig. S15. A layer in the 3D network material that experiences fracture failure under uniaxial tension. The fracture positions are marked by red circles. Scale bar, 500 μm.

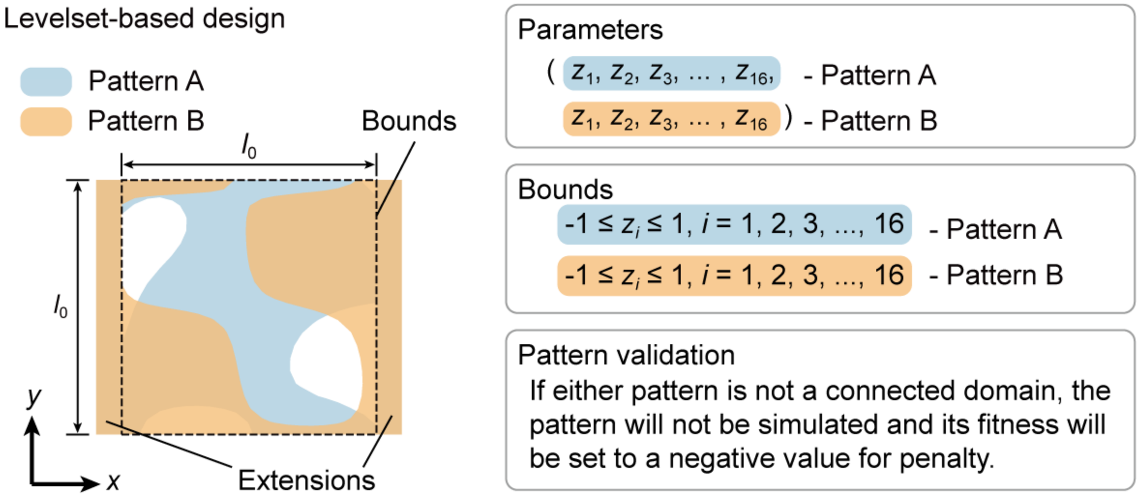

Fig. S16. Parametrization scheme for levelset-based multilayer precursor patterns used for topology optimization. The 'A' and 'B' layers are independently controlled by their corresponding parameters. The levelset function of each layer is in the same form as that in fig. S13.

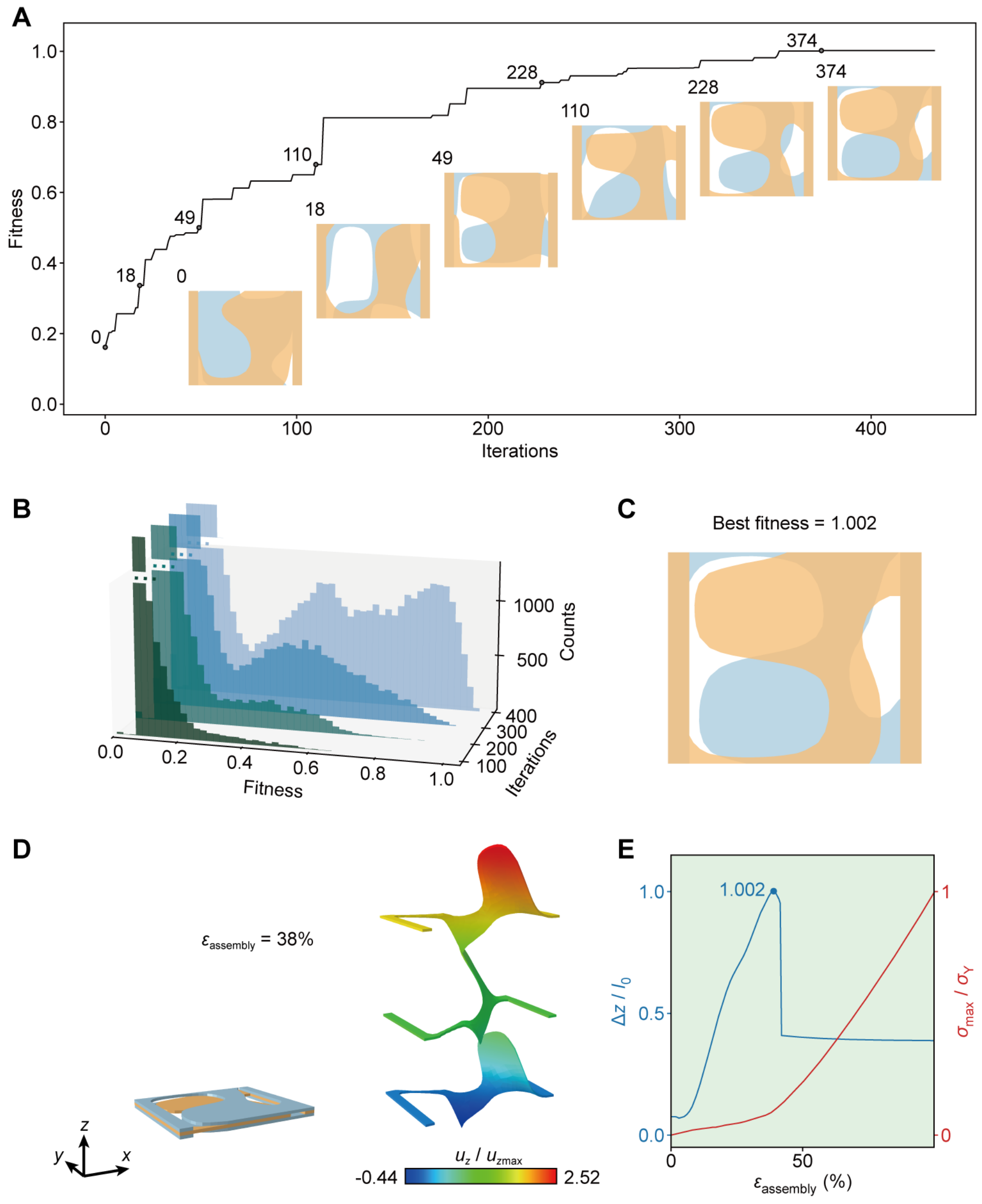

Fig. S17. Multilayer topology optimization of levelset-based precursor designs. (**A**) Fitness evolution curve of the topology optimization, where some key intermediate results during iterations are marked and shown. (**B**) Histogram of the fitness of all valid individuals. (**C**) Optimization result of levelset-based designs. (**D**) Deformed configurations of the optimal 2D precursor under the assembly strain that maximizes its height. (**E**) Relation of the normalized interlayer separation and normalized Mises stress of the structure versus the assembly strain.

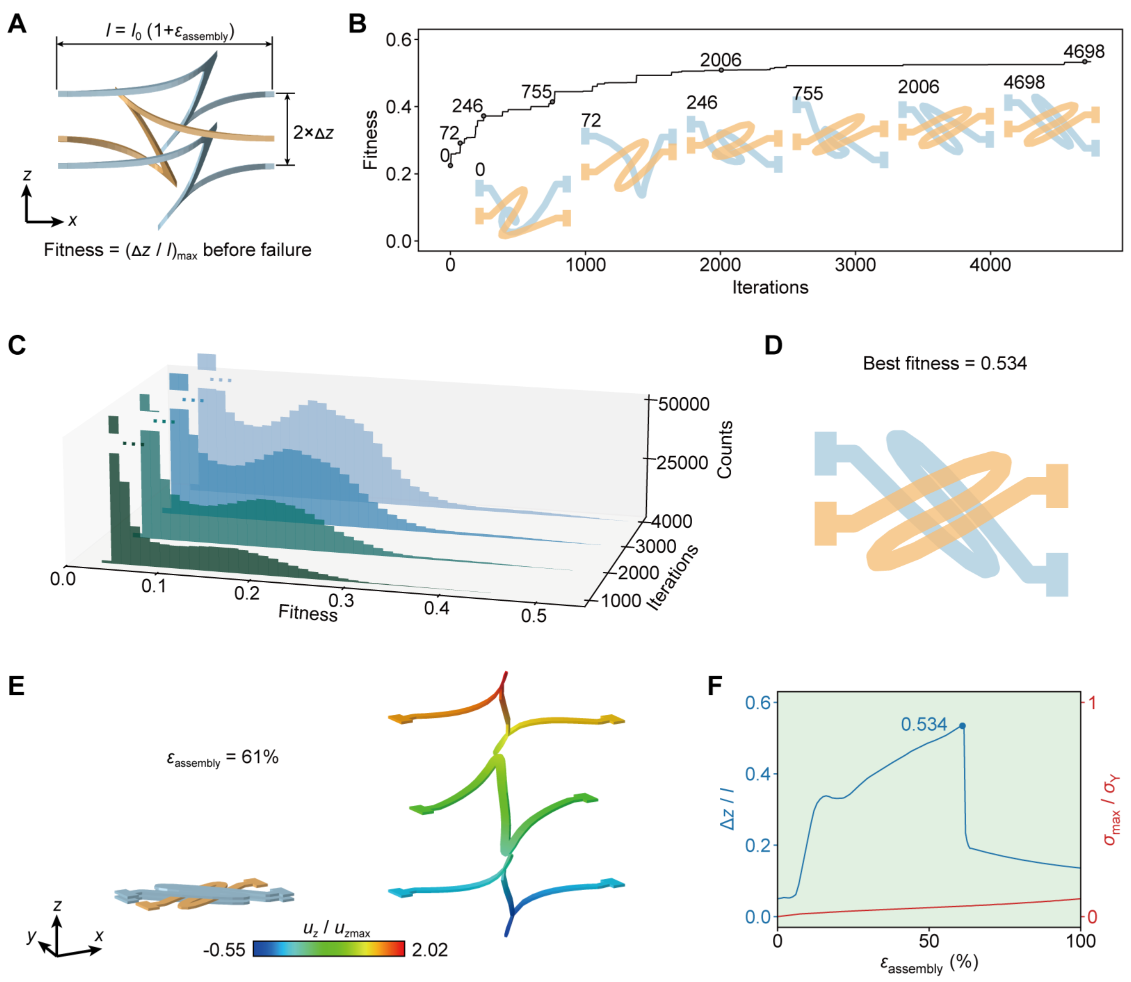

Fig. S18. Multilayer topology optimization for an alternative objective function Δz / l. (**A**) Definition of an alternative objective function to emphasize to emphasize the aspect ratio in the *x*-*z* plane. (**B**) Fitness evolution curve of the topology optimization, where some key intermediate results during iterations are marked and shown. (**C**) Histogram of the fitness of all valid individuals. (**D**) Optimization result based on the alternative objective function. (**E**) Deformed configurations of the optimal 2D precursor under the assembly strain that maximizes its height. (**F**) Relation of the normalized interlayer separation and normalized Mises stress of the structure versus the assembly strain.

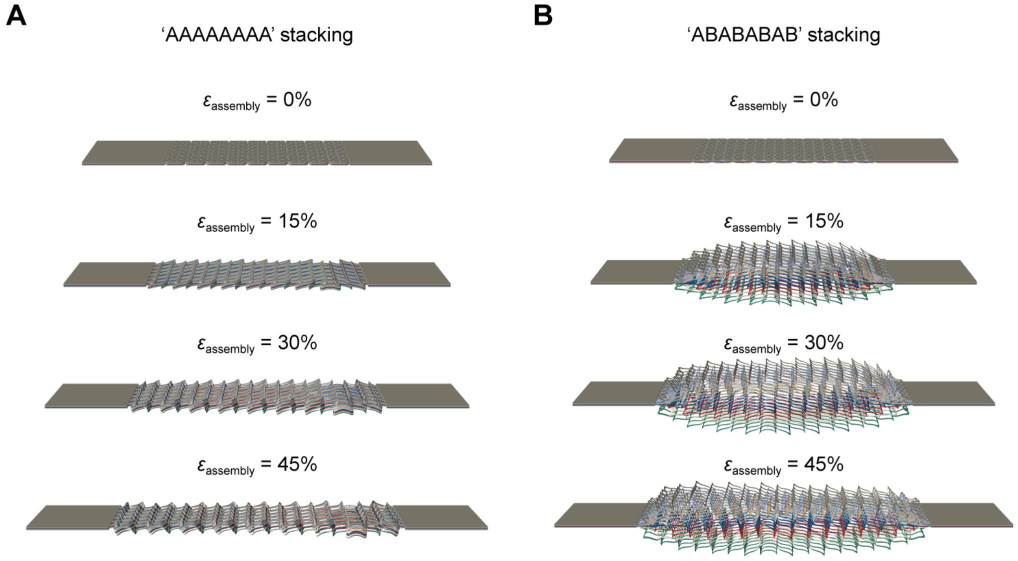

Fig. S19. Comparison between assembled 3D configurations of multilayer precursors without (A) and with (B) alternation between odd and even layers.

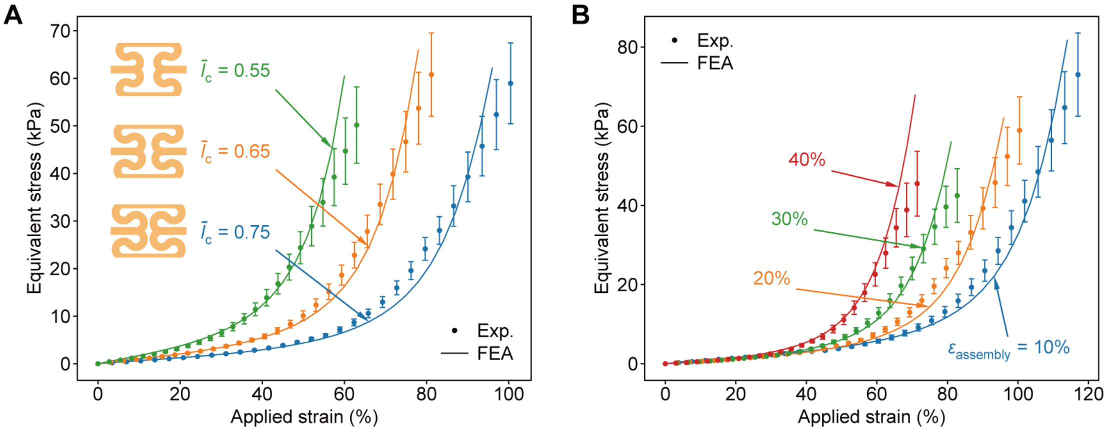

Fig. S20. Effects of key geometric parameters on the J-shaped uniaxial stress-strain curves of the network materials. (**A**) Effect of the effective length ($\bar{l}_c = l_c / l_0$), where the other parameters are fixed as $w / l_0 = 0.1$, $t / l_0 = 0.02$, $l_h / l_0 = 0.7$, and $\varepsilon_{assembly} = 20\%$. (**B**) Effect of the assembly strain ($\varepsilon_{assembly}$), where the other parameters are fixed as $w / l_0 = 0.2$, $t / l_0 = 0.02$, $l_c / l_0 = 0.75$, and $l_h / l_0 = 0.7$.

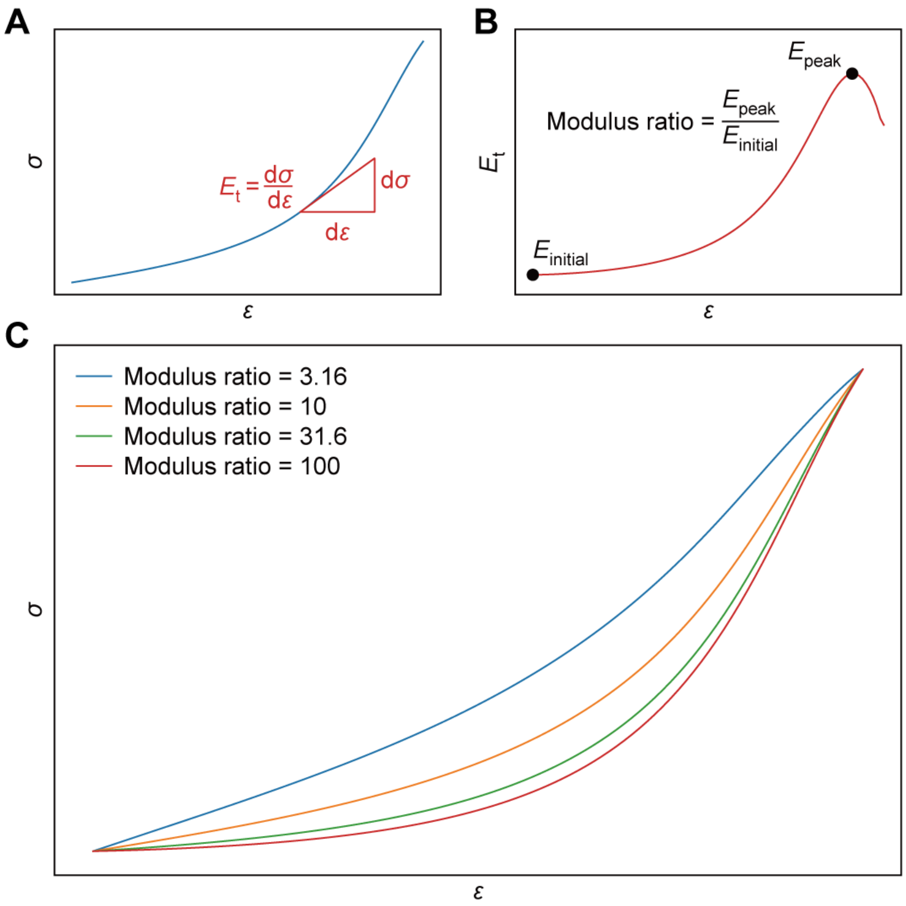

Fig. S21. Definition of the modulus ratio in the J-shaped stress-strain curve. (**A**) Definition of the tangential modulus. (**B**) Definition of the modulus ratio. (**C**) Illustration of stress-strain curves with the same ends but four different modulus ratios.

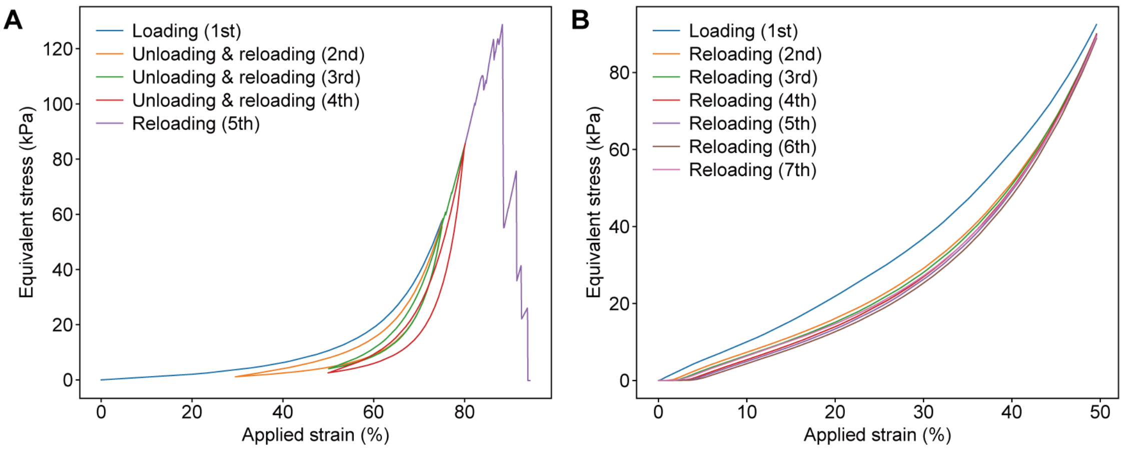

Fig. S22. Mechanical behavior of the 3D network material during loading, unloading and reloading processes. (**A**), Stress-strain curve of the network material during a typical repeated loading/unloading process. (**B**) Stress-strain curve of the network material during multiple loading cycles.

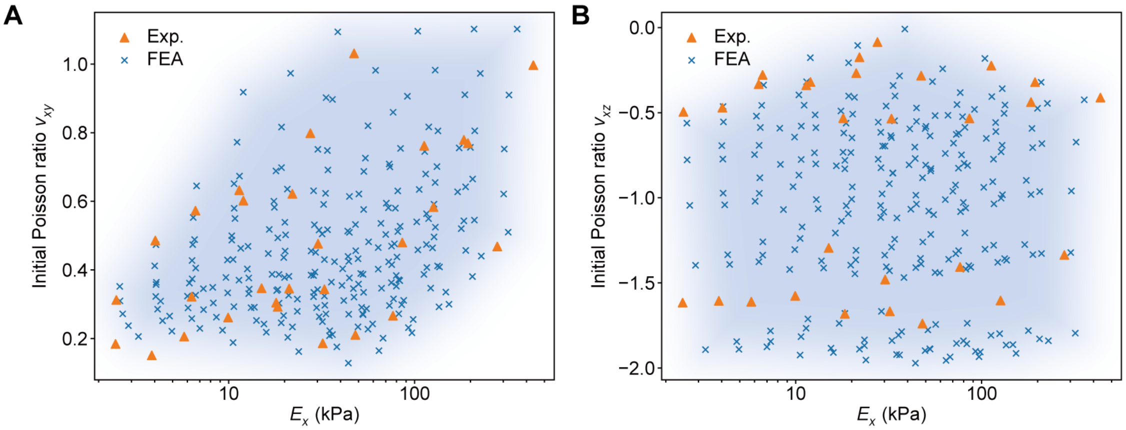

Fig. S23. Feasible region in terms of the Poisson ratio and the equivalent modulus along *x*-axis direction. The experimental and FEA results are marked as data points, and the background color represents the area covered by FEA results. (**A**) Poisson ratio v_{xy} versus the modulus E_x. (**B**) Poisson ratio v_{xz} versus the modulus E_x. The parameters used in the experiments and FEA are in the ranges of [0.05, 0.1] for w / l_0, [0.02, 0.04] for t / l_0, [0.45, 0.75] for l_c / l_0, and [20%, 40%] for $\varepsilon_{assembly}$, noting that l_h / l_0 is fixed as 0.7.

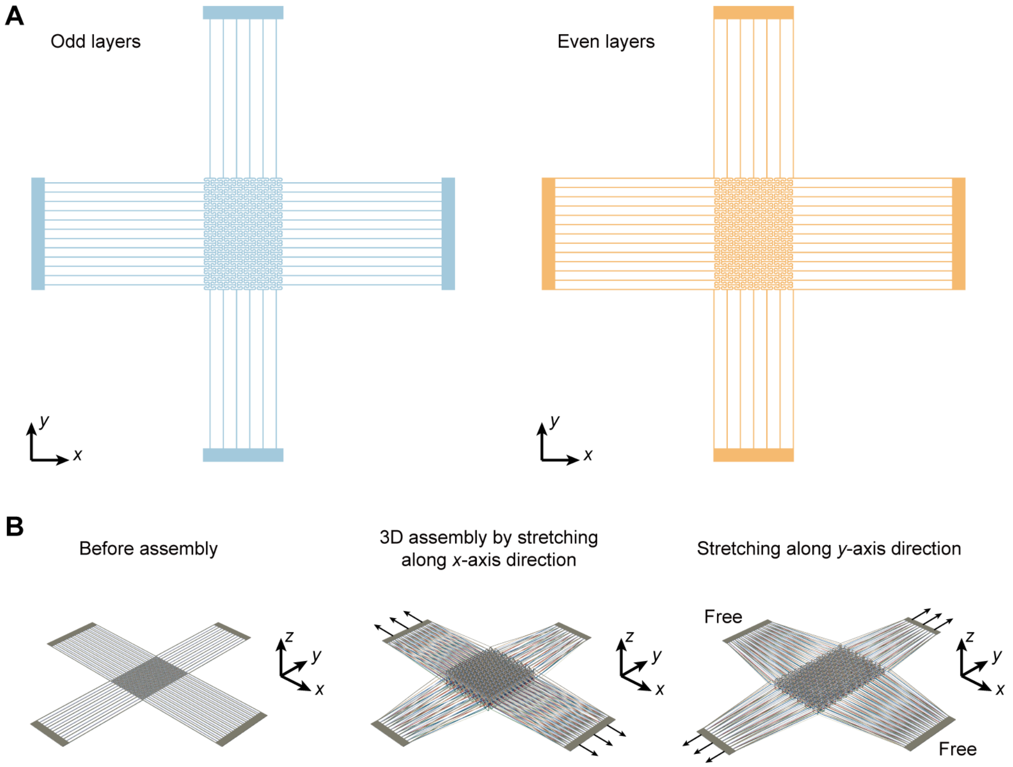

Fig. S24. Tensile test of the 3D network material along the *y*-axis direction. (**A**) Precursor design of the specimen prepared for tensile test along the *y*-axis direction. (**B**) Schematic illustration of the tensile-induced assembly and loading procedure for tensile test along the *y*-axis.

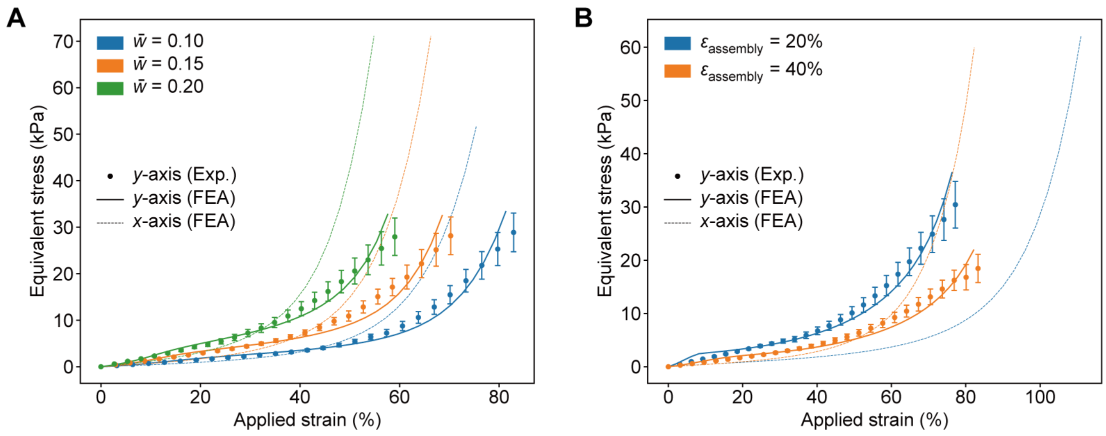

Fig. S25. Effects of key geometric parameters on the J-shaped uniaxial stress-strain curves along the *y*-axis direction. (**A**) Effect of the normalized width (w / l_0), where the other parameters are fixed as $t / l_0 = 0.02$, $l_c / l_0 = 0.65$, $l_h / l_0 = 0.7$, and $\varepsilon_{assembly} = 40\%$. (**B**) Effect of the assembly strain ($\varepsilon_{assembly}$), where the other parameters are fixed as $w / l_0 = 0.15$, $t / l_0 = 0.02$, $l_c / l_0 = 0.75$, and $l_h / l_0 = 0.7$.

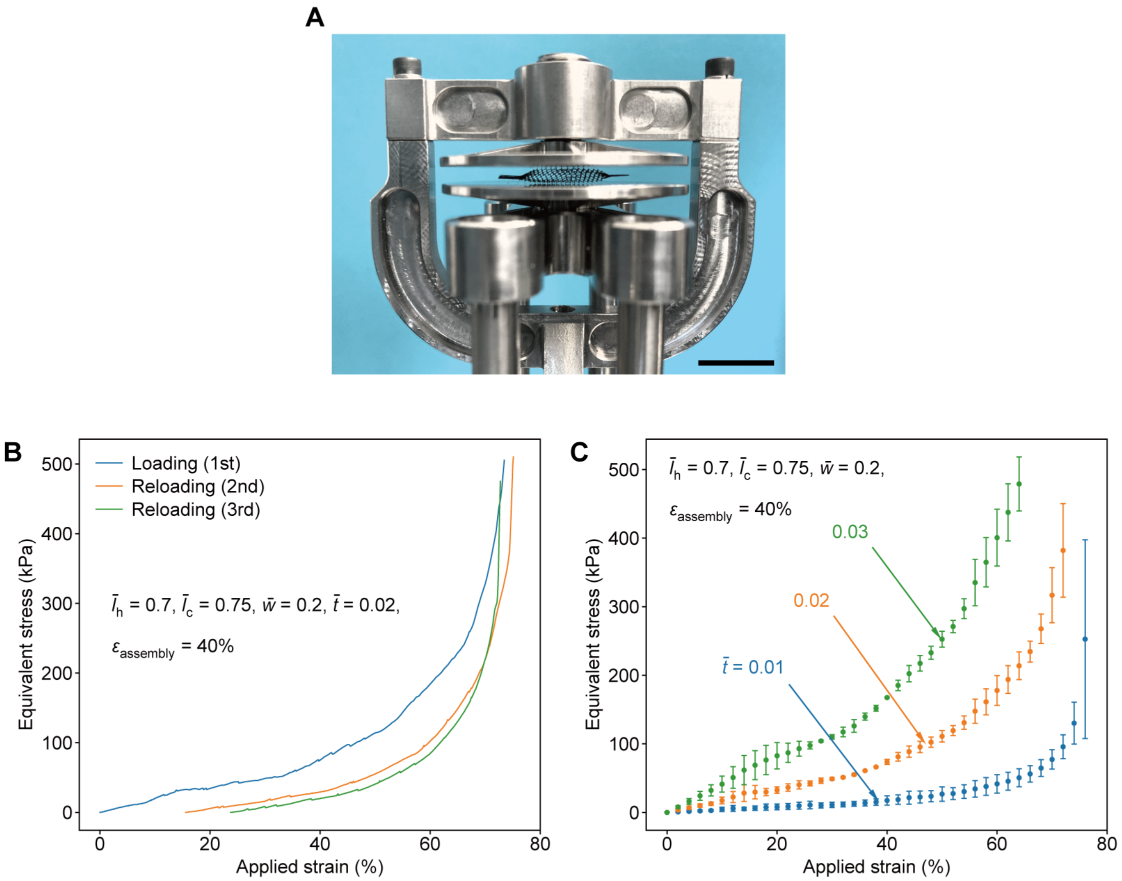

Fig. S26. Mechanical behavior of the 3D network material under the *z*-axis compression. (**A**) Experimental setup for the *z*-axis compression. Scale bar, 2 cm. (**B**) Stress-strain curve of the network material in three loading cycles. (**C**) Effect of the normalized thickness (t / l_0) of the precursors on the stress-strain curve of network material during the *z*-axis compression. The parameters of the samples are labeled in the corresponding panels, where $\bar{l}_h = l_h / l_0$ denotes the normalized length of the basic unit cell in the *y*-axis direction. The error bars represent the standard deviation based on measurements from 3 individual specimens.

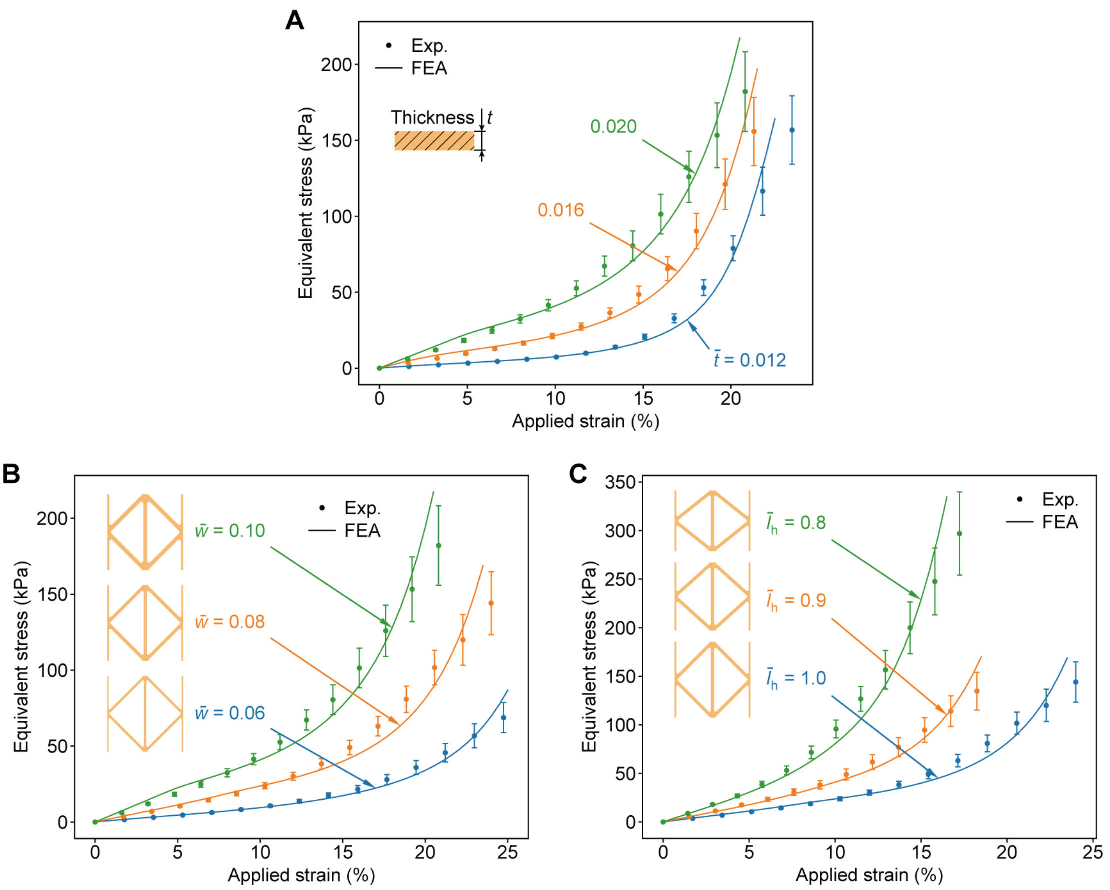

Fig. S27. Mechanical properties of the 3D network material assembled from precursors with the design shown in fig. S7. (**A**) Effect of the normalized thickness (t / l_0), where the other parameters are fixed as w / l_0 = 0.1, l_h / l_0 = 1, and $\varepsilon_{assembly}$ = 4%. (**B**) Effect of the normalized width (w / l_0), where the other parameters are fixed as t / l_0 = 0.02, l_h / l_0 = 1, and $\varepsilon_{assembly}$ = 4%. (**C**) Effect of the normalized basic unit cell length (l_h / l_0) along the y-axis direction, where the other parameters are fixed as w / l_0 = 0.08, t / l_0 = 0.02, and $\varepsilon_{assembly}$ = 4%.

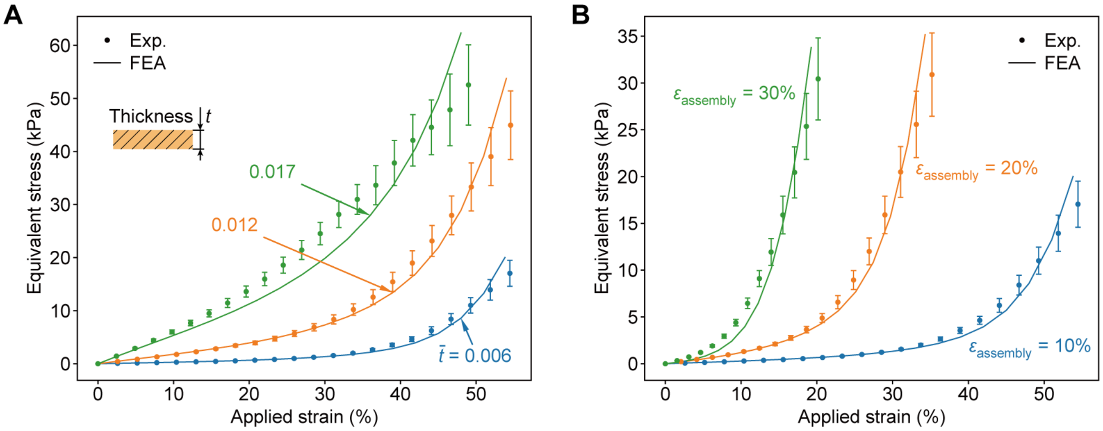

Fig. S28. Mechanical properties of the 3D network material assembled from precursors with the design shown in in fig. S8. (**A**) Effect of the normalized thickness (t / l_0), where the other parameters are fixed as l_h / l_0 = 1.59, l_c / l_0 = 1.16, w / l_0 = 0.15, w_{cut} / l_0 = 0.023, and $\varepsilon_{assembly}$ = 10%. (**B**) Effect of the assembly strain ($\varepsilon_{assembly}$), where the other parameters are fixed as l_h / l_0 = 1.59, l_c / l_0 = 1.16, w / l_0 = 0.15, w_{cut} / l_0 = 0.023, t / l_0 = 0.006, and $\varepsilon_{assembly}$ = 10%.

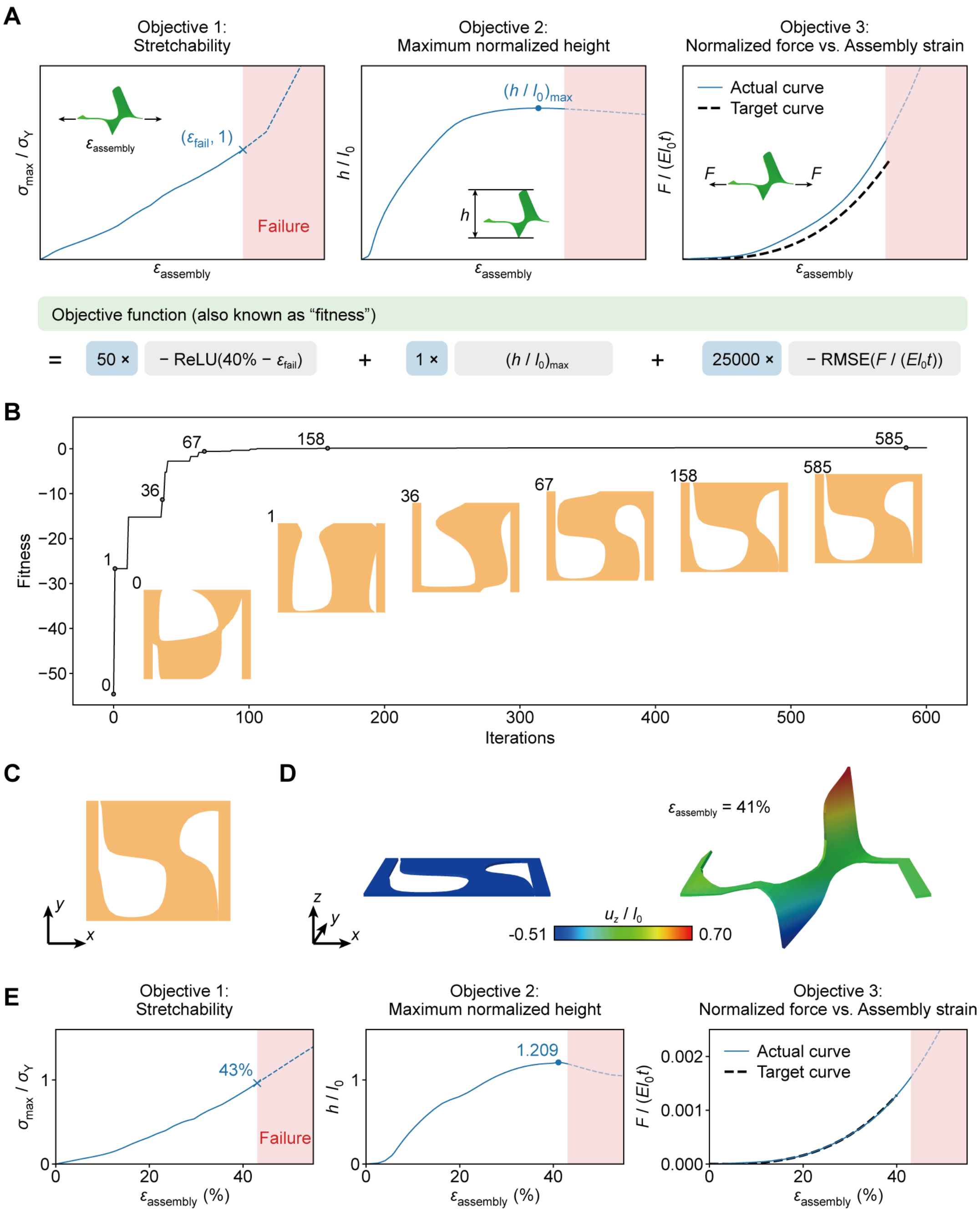

Fig. S29. Multi-objective optimization of single-layer structure with levelset-based pattern designs. (**A**) Illustration of the three optimization objectives and the definition of the objective function. (**B**) Fitness evolution curve of the topology optimization, with key intermediate results marked. (**C**) Optimization result of the 2D precursor. (**D**) Deformed configurations of the optimal 2D precursor under the assembly strain that maximizes its height. (**E**) Relationship between relative Mises stress, maximum normalized height, and normalized force of the optimal structure and the assembly strain.

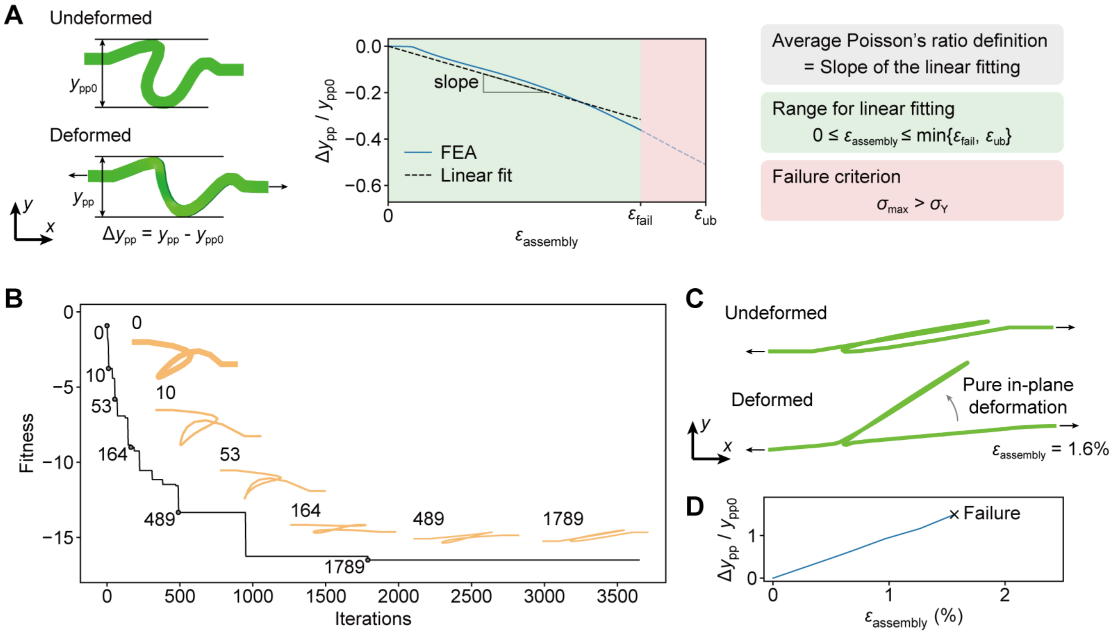

Fig. S30. Topology optimization for in-plane Poisson's ratio of single-layer structure with ribbon-based pattern design. (**A**) Definition of the objective function (i.e., the average Poisson's ratio) in the topology optimization. (**B**) Fitness evolution curve of the topology optimization, with key intermediate results marked. (**C**) Initial and deformed configuration of the structure with the optimal pattern design. (**D**) Relationship of normalized *y*-axial span increment versus assembly strain for the optimal structure.

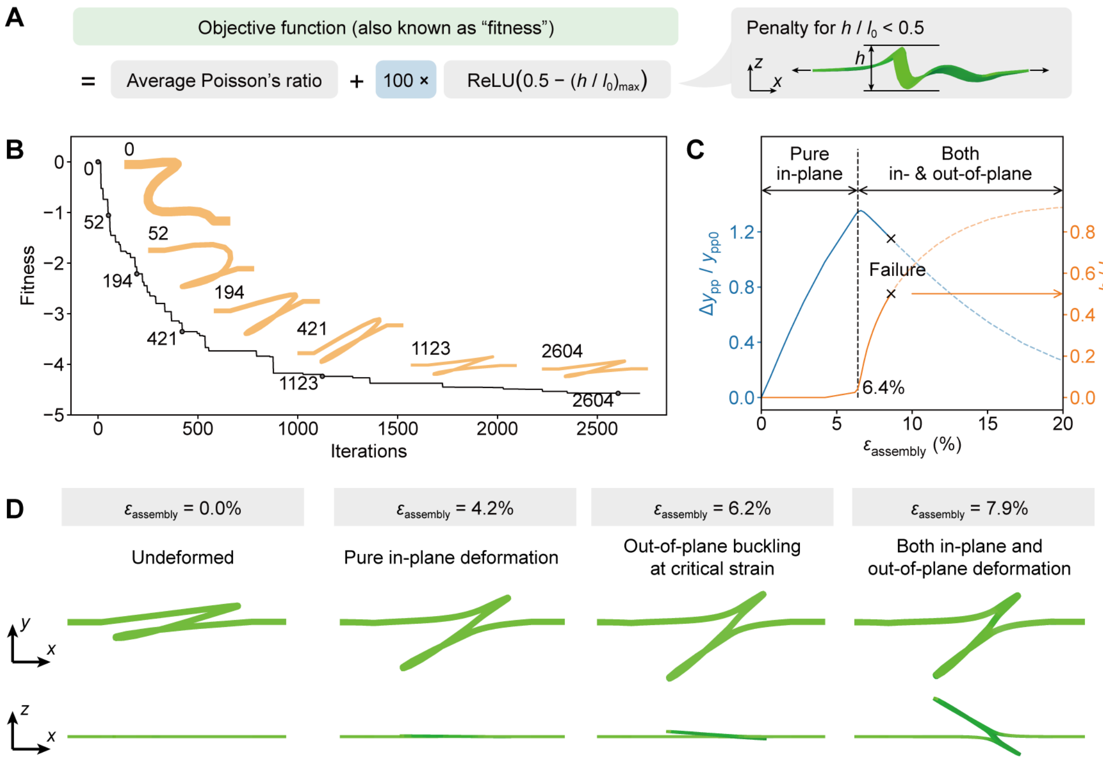

Fig. S31. Topology optimization considering both in-plane Poisson's ratio and out-of-plane deformation for single-layer structure with ribbon-based pattern design. (**A**) Definition of the objective function that considers both in-plane Poisson's ratio and out-of-plane deformation. (**B**) Fitness evolution curve of the topology optimization, with key intermediate results marked. (**C**) Relationship of the normalized y-axial span increment and normalized height versus assembly strain for the optimal structure. (**D**) Deformation process of the optimal structure.

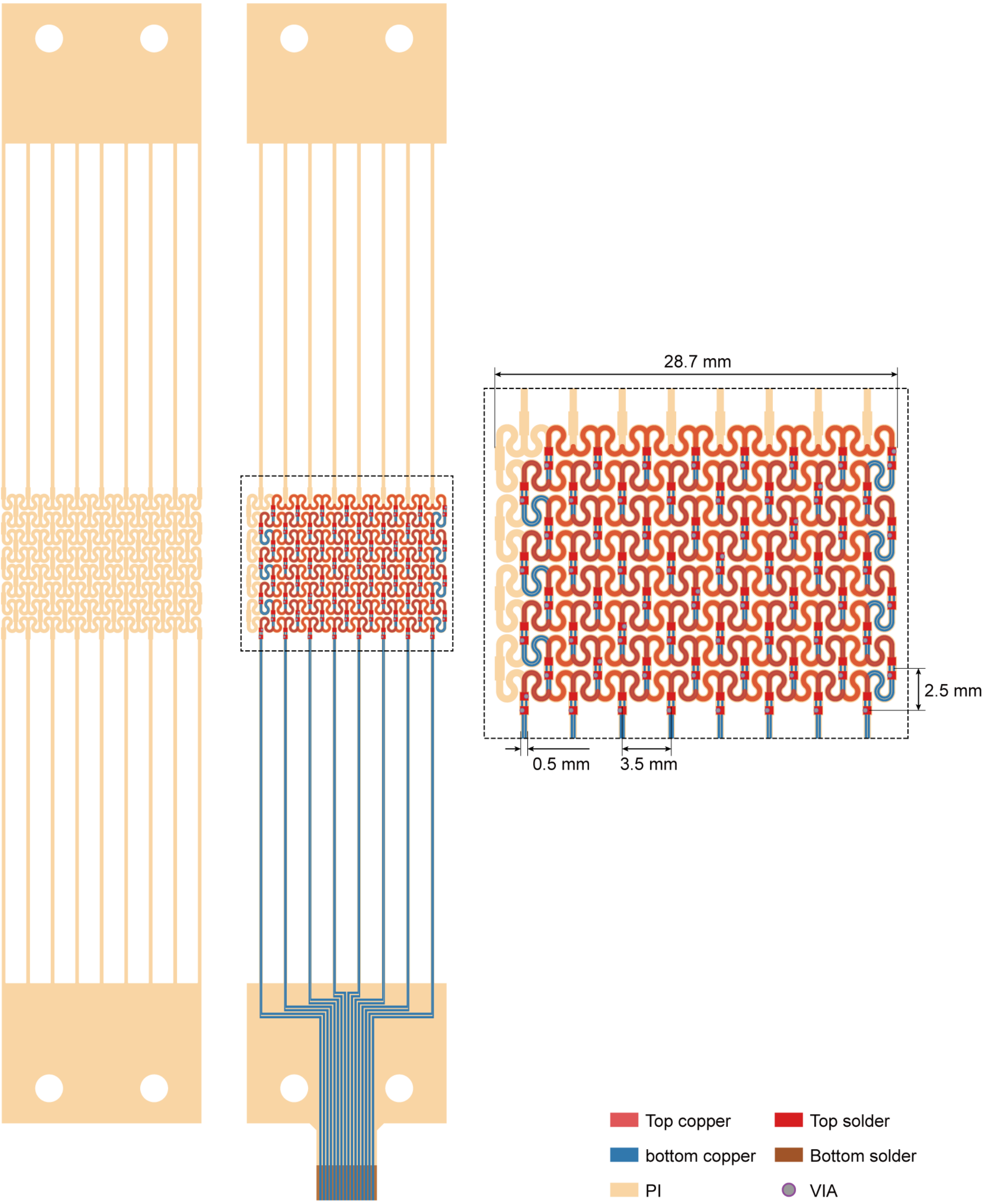

Fig. S32. Schematic diagram of the precursors of the volumetric 3D display device.

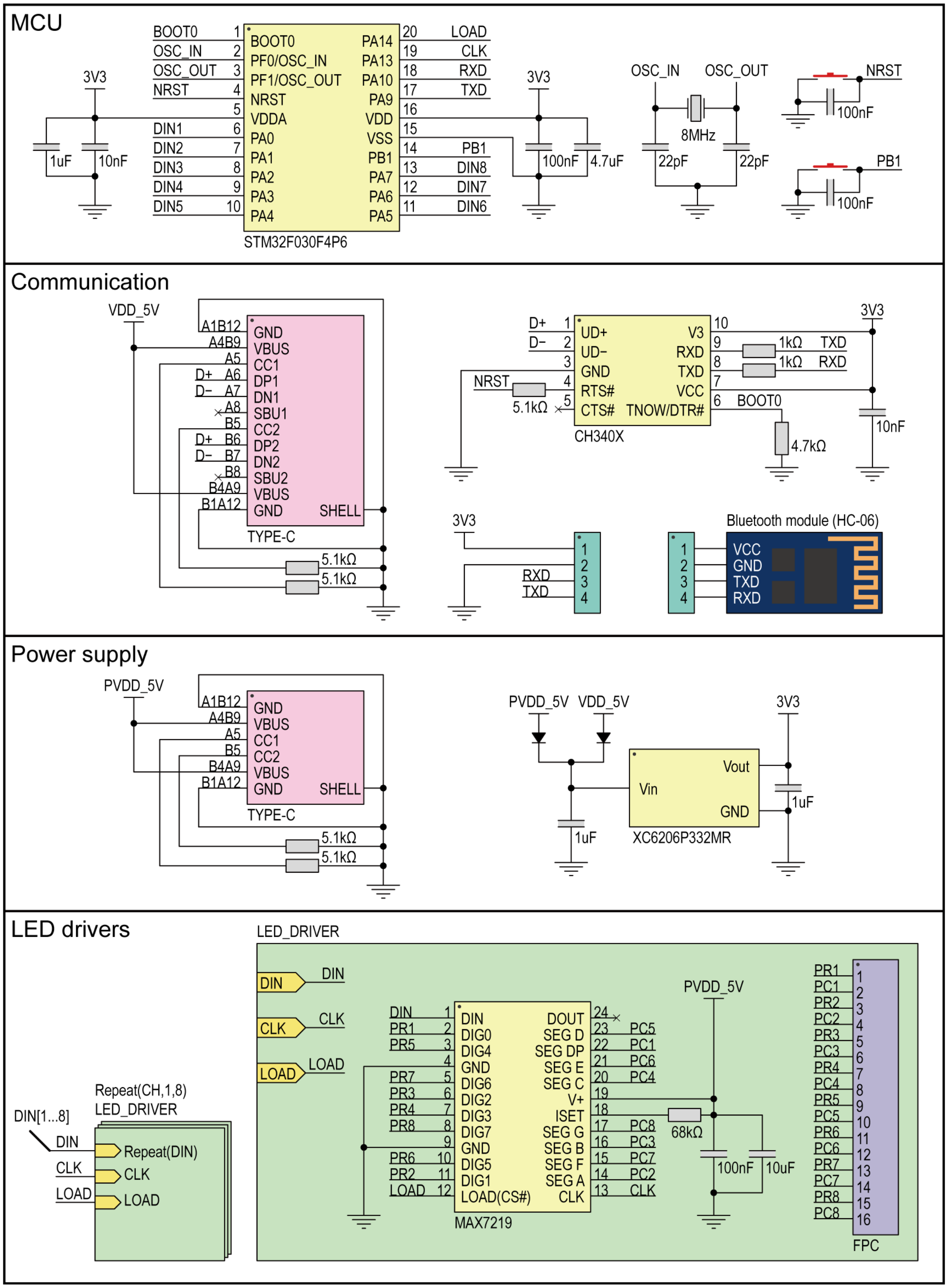

Fig. S33. Schematic diagram of the LED controller circuit.

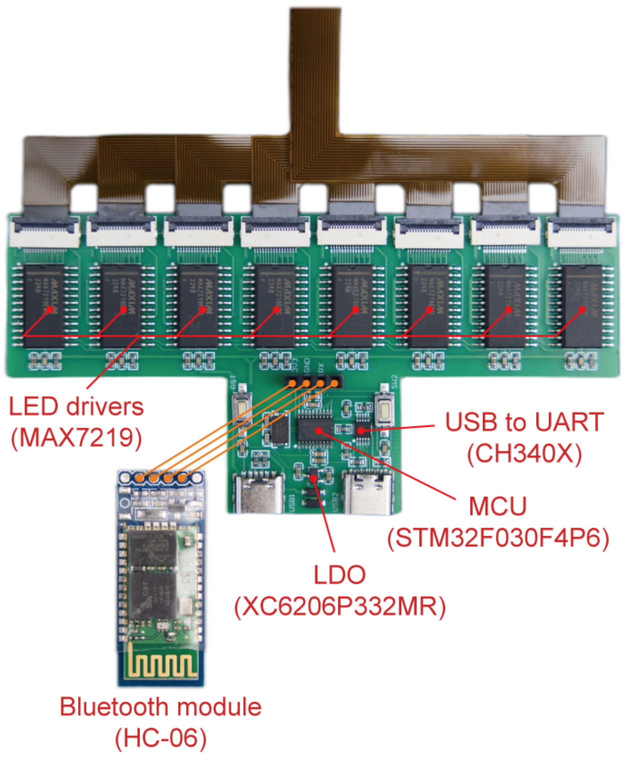

Fig. S34. PCB diagram of the LED controller. The model/type of all utilized chips are labeled in this diagram.

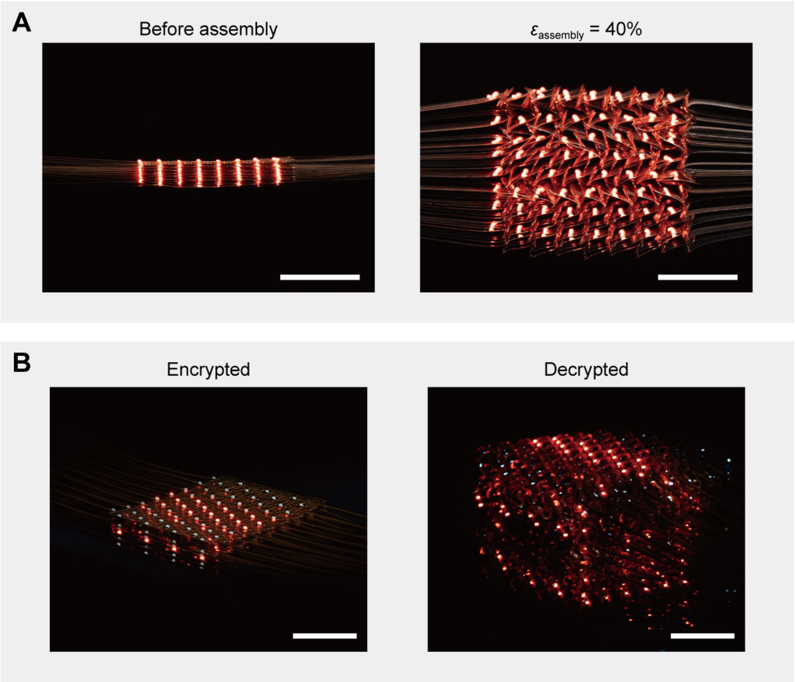

Fig. S35. Demonstrations of the volumetric 3D display based on the precursor design with in-plane dimensions scaled by a factor of two in comparison to that shown in Fig. 6 (A to H). (**A**) Comparison of the device configurations before and after assembly. (**B**) Demonstration of information decryption, where the two images correspond to the states before and after assembly with 40% strain. Scale bars, 2 cm.

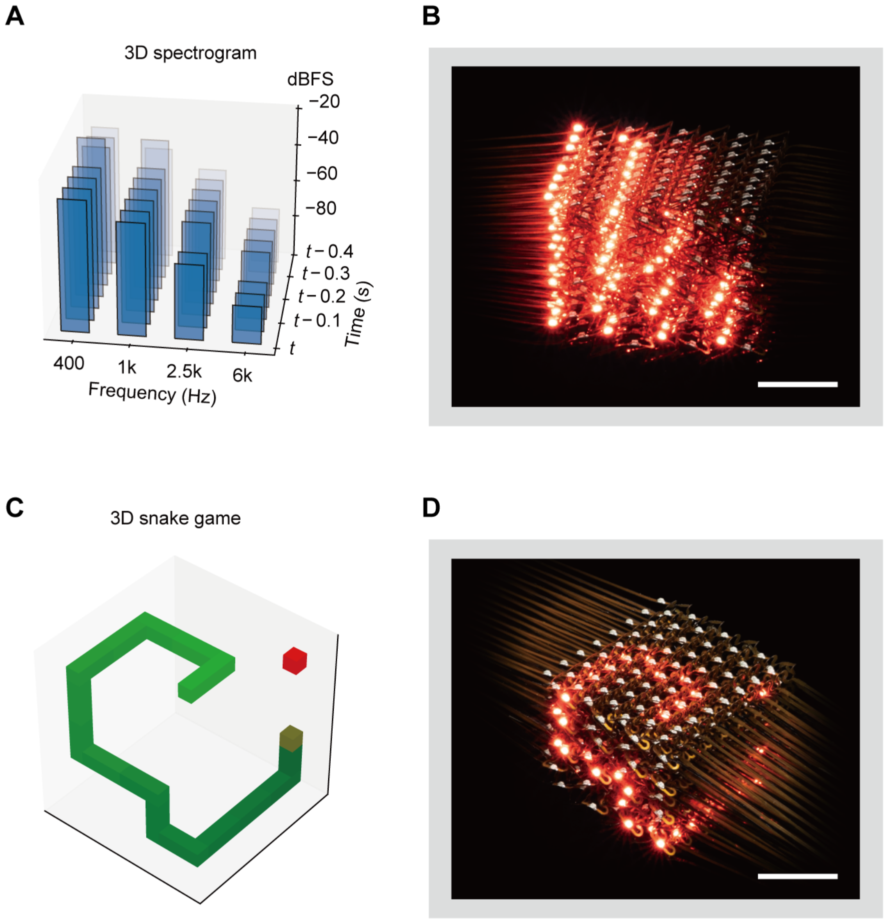

Fig. S36. Applications based on the volumetric 3D display device. (**A**) Schematic illustration of a 3D spectrogram, which displays the power spectral density of four typical frequency ranges. The displayed spectrogram updates in real time, while the old spectrums continue to flow backwards. (**B**) Visual effect of the 3D spectrum displayed on the 3D device. (**C**) Schematic illustration of a snake game in 3D space, where a pixelated snake keeps chasing food while preventing collisions with walls and its own body. (**D**) Visual effect of the 3D snake game displayed on the 3D device. Scale bar, 1 cm.

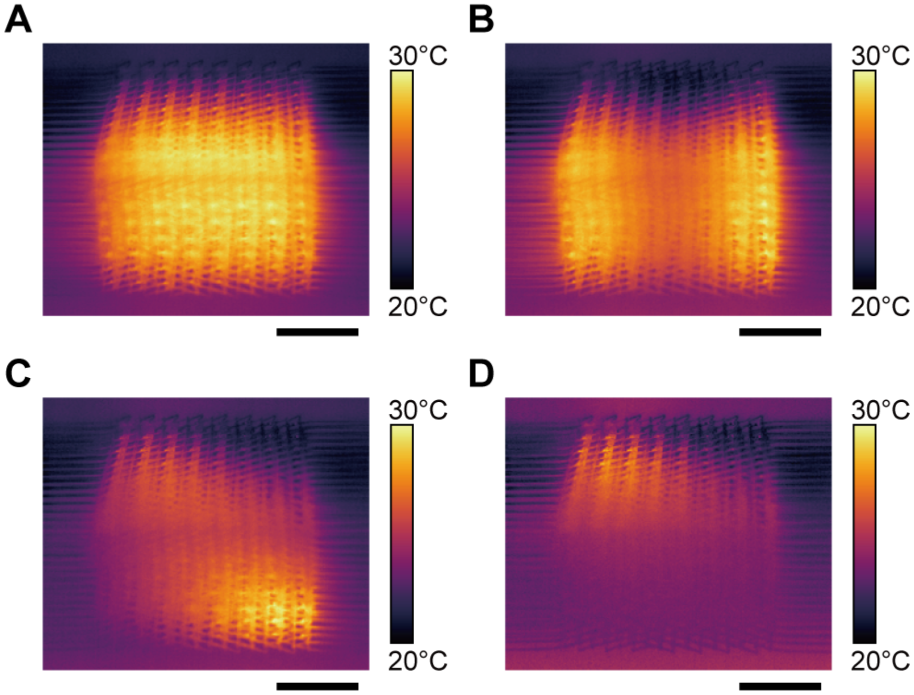

Fig. S37. Different 3D temperature distributions created by leveraging the thermal effect of the 3D LED array. (**A**) Heat uniformly distributed in the central region. (**B**) Heat distributed at the left and right boundaries. (**C**) Heat distributed near the diagonal. (**D**) Heat distributed at a corner. Scale bars, 1 cm.

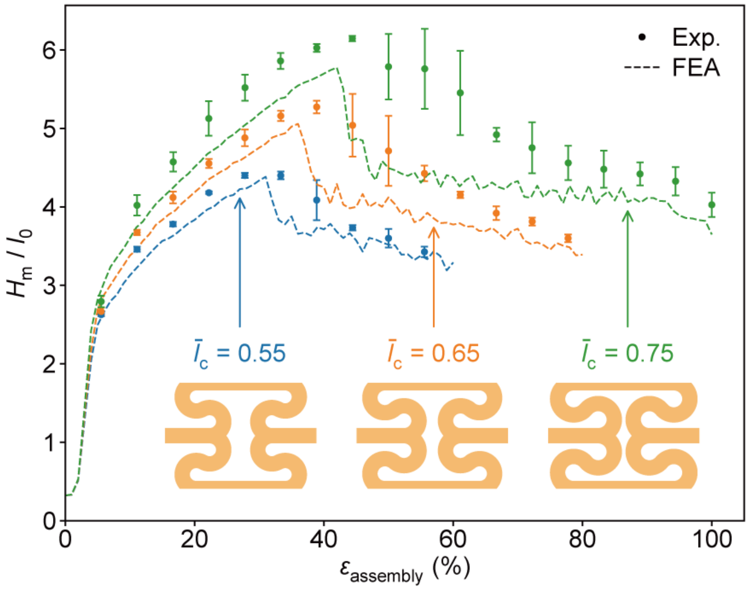

Fig. S38. Effect of the normalized effective length (l_c / l_0) on the normalized height (H_m / l_0) of the multilayered structure. The other parameters are fixed as w / l_0 = 0.1, t / l_0 = 0.02, and l_h / l_0 = 0.7. Note that the FEA results presented here do not take account of friction between adjacent layers, resulting in the collapse occurring slightly earlier than that observed in the experiments.

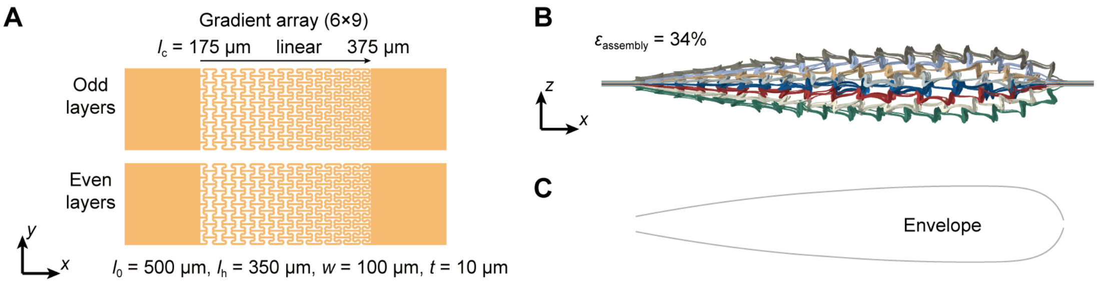

Fig. S39. Assembly of multilayer network structure with gradient pattern design. (**A**) Geometric design of the multilayer precursors. (**B**) Deformed configuration of the network structure under stretching. (**C**) Teardrop-shaped envelope of the network structure.

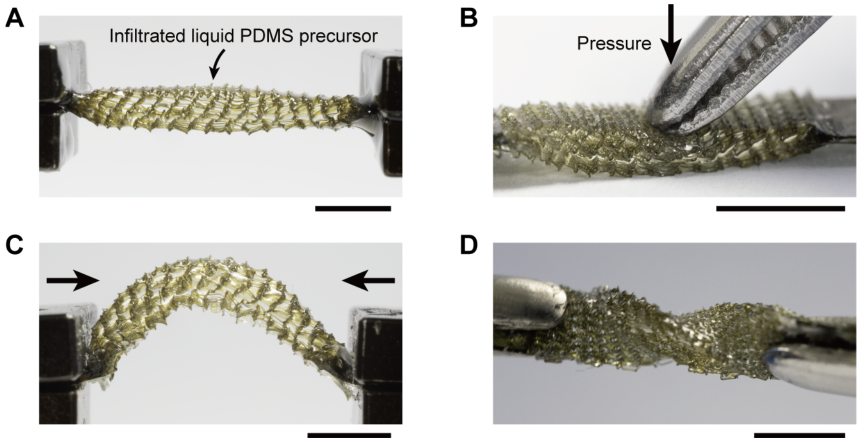

Fig. S40. Improvement of structural integrity via PDMS encapsulation of the 3D network structure. (**A**) Network material fully infiltrated with liquid PDMS precursor. (**B**) Encapsulated network material compressed by a tweezer. (**C**) Encapsulated network material under *x*-axial compression. (**D**) Encapsulated network material subjected to large torsional deformation. Scale bars, 5 mm.

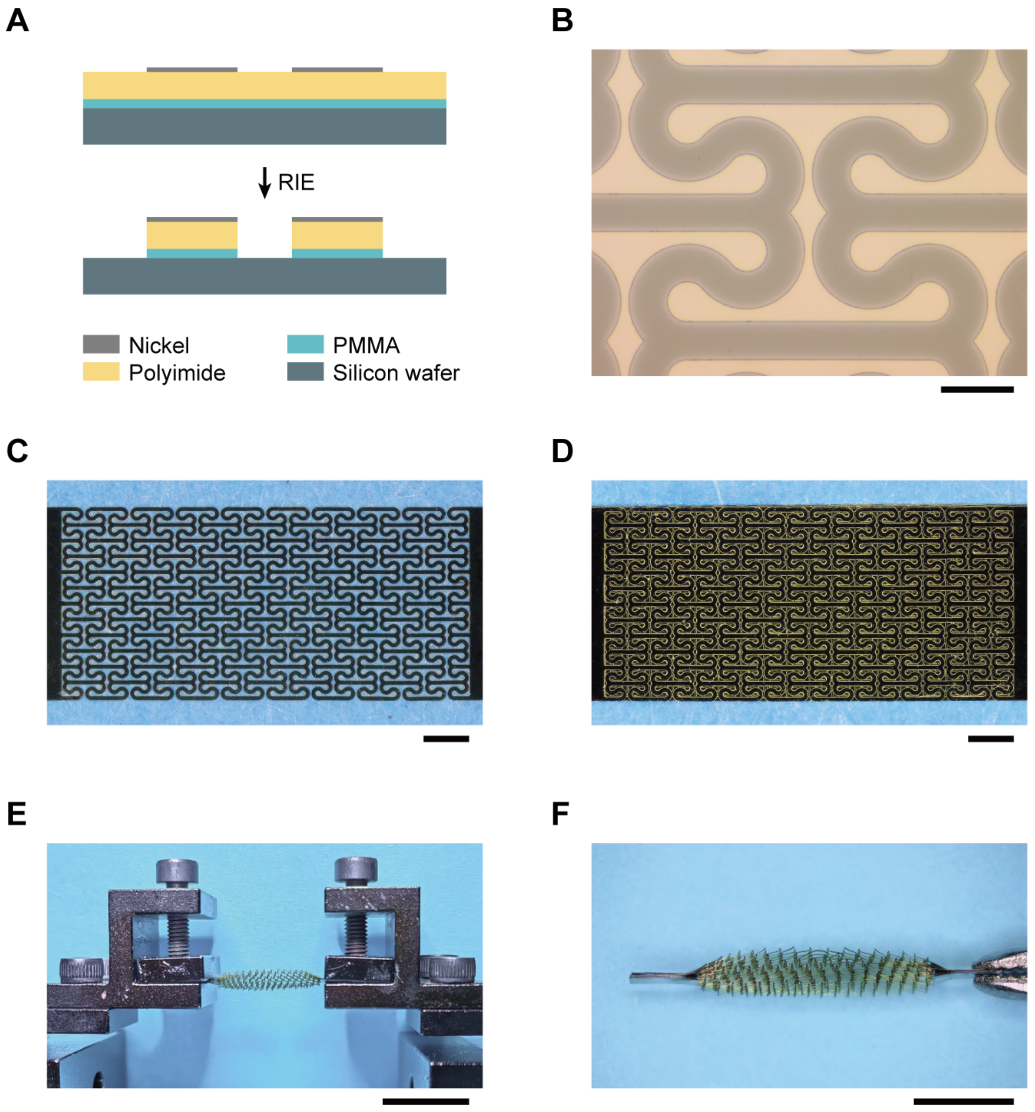

Fig. S41. Fabrication of the 3D network material. (**A**) Illustration of the manufacturing of the planar precursors. (**B**) Optical image of the precursors right after the photolithography. Scale bar, 100 μm. (**C**) A single layer of planar precursor. Scale bar, 1 mm. (**D**) Multilayer planar precursors stacked by transfer printing. Scale bar, 1 mm. (**E**) Assembled multilayer structure on the loading platform. Scale bar, 1 cm. (**F**) Freestanding 3D network material obtained through thermoforming. Scale bar, 5 mm.

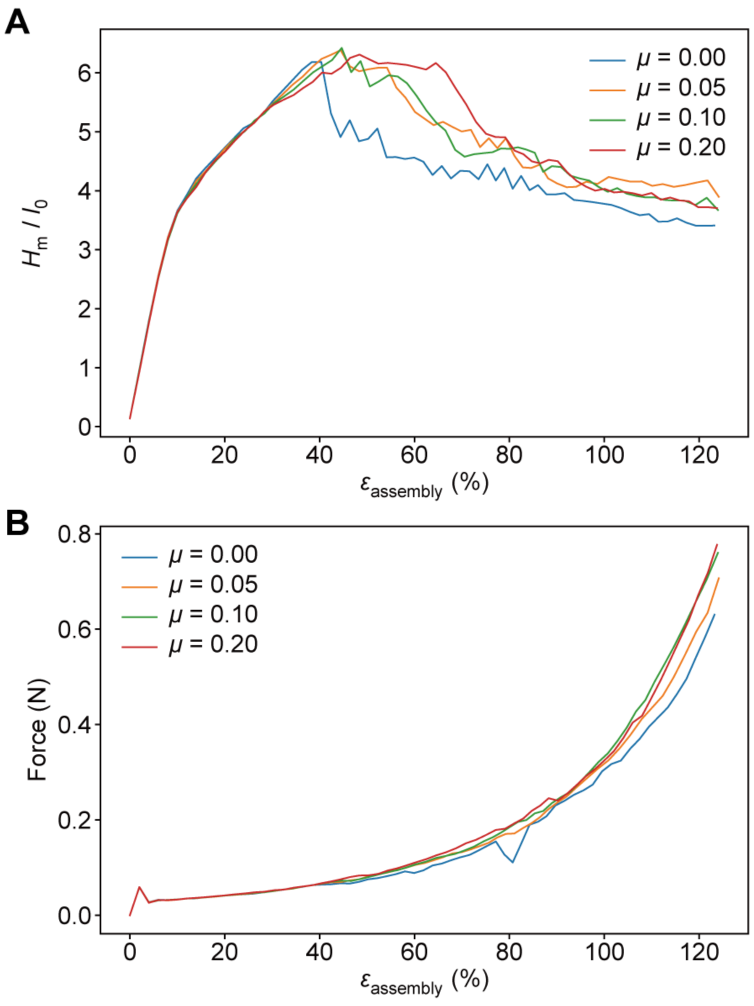

Fig. S42. Effects of frictional coefficient *μ* on the normalized total height (A) and force responses of the multilayer precursors during assembly (B). The curves are obtained from the FEA of the specimen presented in Fig. 1C with different frictional coefficients.

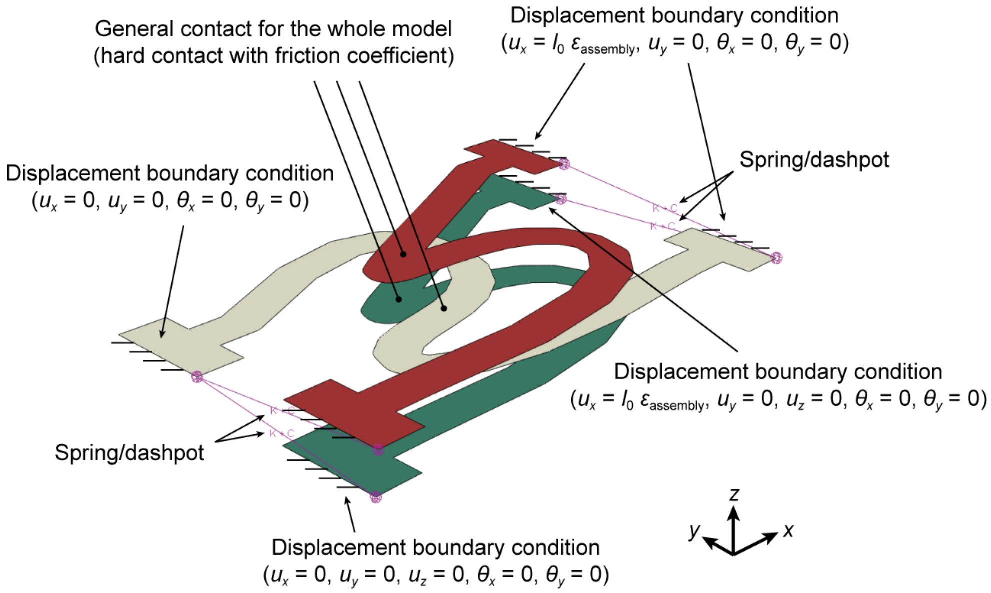

Fig. S43. Finite element model used to simulate the 3D assembly process.

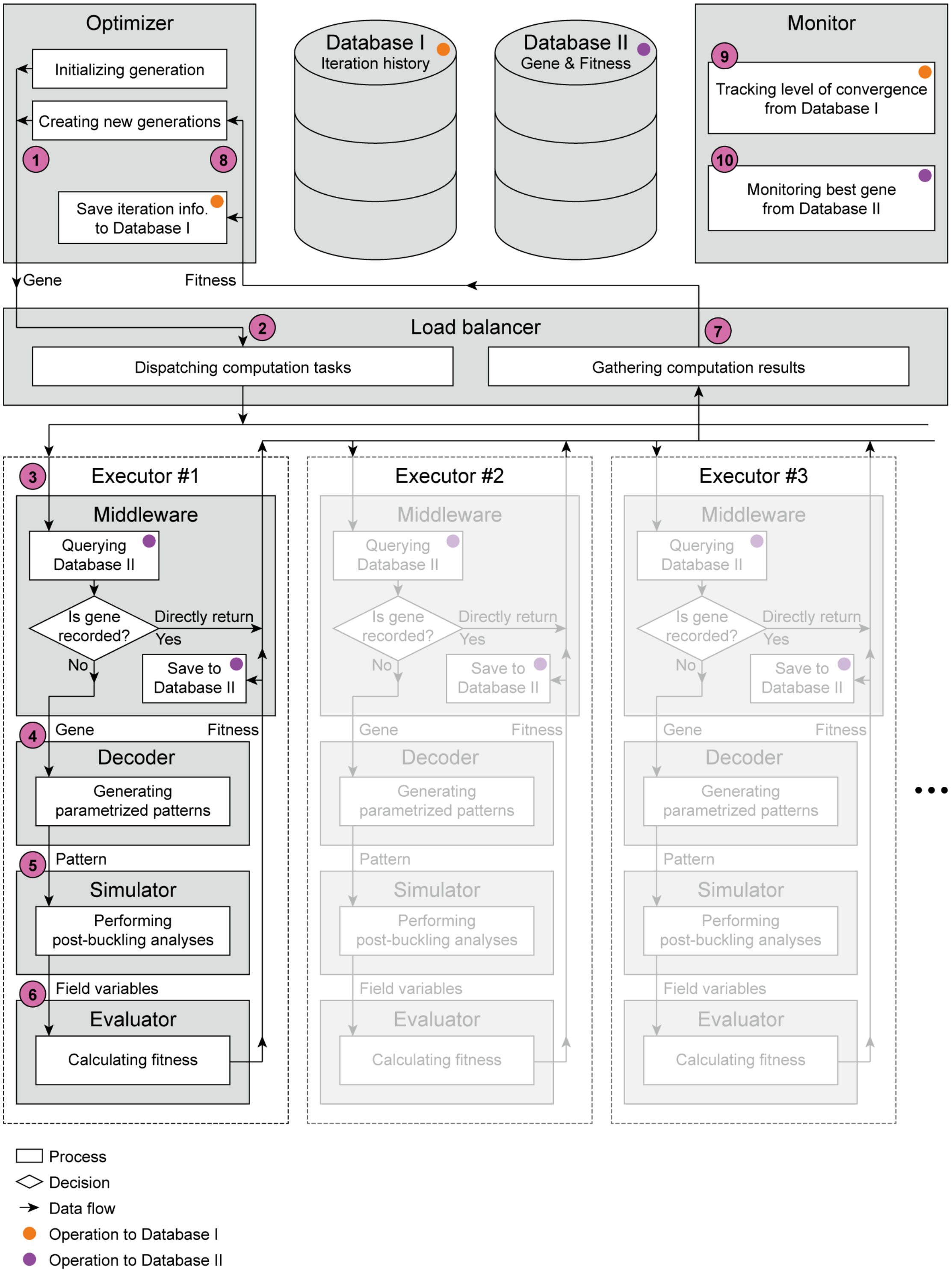

Fig. S44. Architecture of the topology optimization program.

Optimization task	Number of optimization parameters	Total number of simulations	Total number of iterations	Total time consumption (core-hour)	Average time cost of each simulation (core-minute)
Single layer, ribbon-based	11	27,362	168	500	1.10
Single layer, kirigami-type	12	36,890	271	1,000	1.63
Single layer, levelset-based	16	76,861	508	1,800	1.41
Three layers, ribbon-based	20	207,230	727	9,300	2.69
Three layers, levelset-based	32	90,799	433	8,900	5.88
Three layers, ribbon-based, alternative objective	20	1,355,830	4,740	60,500	2.68

Table S1. Time consumption of the optimization tasks.

Category	Technology	Advantages	Disadvantages
Holography	Digital holography(*70, 71*)	Ultra-high resolution, and capability to accurately replicate real light fields	Limited viewing angles, scale constraints, and requirement of sophisticated spatial light modulators
Light field display	Layer-based(*77*)	High resolution, and rapid refresh rates	Trade-off between resolution and wide perspective
	Projector-based(*78*)		
	Integral imaging(*79*)		
Volumetric 3D display	Swept-volume display(*80*)	High refresh rates, wide viewing angles, and cost-effective	Requirement of mechanical parts, and bulky in overall size
	Solid-volume display(*81*)	Non-uniformity in resolution along different directions	Limited viewing angles
	Controlled particles-based(*74, 75*)	Free-space display, and no screen required	Low refresh rates, limited scalability, and sensitive to environmental disturbances
	This work	High refresh rates, wide viewing angles, and reconfigurability	Relatively lower resolution compared to other advanced technologies

Table S2. Comparison of the developed volumetric 3D display with other glasses-free 3D display technologies.

Movie S1. Experimental results on the assembly of the 3D network material through tensile buckling.

Movie S2. FEA results on the assembly of the 3D network material and the deformation of its unit cell. The deformation of the network materials during the assembly is demonstrated from three different perspectives.

Movie S3. Evolution of precursor patterns throughout the multilayer topology optimization. The video visualizes the multilayer topology optimization based on the ribbon-based scheme. It shows the progression of the population across iterations, the best individual up to each generation, and the evolution of the best fitness value versus iteration number.

Movie S4. Demonstration of various 3D patterns and applications based on the volumetric 3D display. The video presents a series of 3D animations, beginning with a breathing cube exhibiting fluctuating brightness, followed by a flickering cube. Then, it shows a cubic frame expanding and contracting, and a tetrahedron rotating in the 3D space. The video also includes 3D scrolling text on three faces of a cube. Additionally, the video showcases a static 3D pattern that appears as the letter 'O' from the front and the letter 'X' from the top. Finally, the video demonstrates applications of a 3D snake game and a dynamic 3D spectrogram.

Movie S5. Demonstration of information decryption using the reconfigurable volumetric 3D display. An animation of an athlete running rightward is played on the reconfigurable volumetric 3D display. The profile of the running athlete is difficult to identify in the stress-free state, but becomes easily recognizable after the display is stretched and transformed into the final 3D configuration.

REFERENCES AND NOTES

1. Y. Tang, R. Ballarini, M. J. Buehler, S. J. Eppell, Deformation micromechanisms of collagen fibrils under uniaxial tension. *J. R. Soc. Interface* **7**, 839–850 (2010).

2. A. Gautieri, S. Vesentini, A. Redaelli, M. J. Buehler, Hierarchical structure and nanomechanics of collagen microfibrils from the atomistic scale up. *Nano Lett.* **11**, 757–766 (2011).

3. J. R. Greer, V. S. Deshpande, Three-dimensional architected materials and structures: Design, fabrication, and mechanical behavior. *MRS Bull.* **44**, 750–757 (2019).

4. N. A. Traugutt, D. Mistry, C. Luo, K. Yu, Q. Ge, C. M. Yakacki, Liquid-crystal-elastomer-based dissipative structures by digital light processing 3D printing. *Adv. Mater.* **32**, 2000797 (2020).

5. D. Yan, J. Chang, H. Zhang, J. Liu, H. Song, Z. Xue, F. Zhang, Y. Zhang, Soft three-dimensional network materials with rational bio-mimetic designs. *Nat. Commun.* **11**, 1180 (2020).

6. Y. Wang, L. Li, D. Hofmann, J. E. Andrade, C. Daraio, Structured fabrics with tunable mechanical properties. *Nature* **596**, 238–243 (2021).

7. Y. Ling, W. Pang, J. Liu, M. Page, Y. Xu, G. Zhao, D. Stalla, J. Xie, Y. Zhang, Z. Yan, Bioinspired elastomer composites with programmed mechanical and electrical anisotropies. *Nat. Commun.* **13**, 524 (2022).

8. K.-I. Jang, H. U. Chung, S. Xu, C. H. Lee, H. Luan, J. Jeong, H. Cheng, G.-T. Kim, S. Y. Han, J. W. Lee, J. Kim, M. Cho, F. Miao, Y. Yang, H. N. Jung, M. Flavin, H. Liu, G. W. Kong, K. J. Yu, S. I. Rhee, J. Chung, B. Kim, J. W. Kwak, M. H. Yun, J. Y. Kim, Y. M. Song, U. Paik, Y. Zhang, Y. Huang, J. A. Rogers, Soft network composite materials with deterministic and bio-inspired designs. *Nat. Commun.* **6**, 6566 (2015).

9. K.-I. Jang, K. Li, H. U. Chung, S. Xu, H. N. Jung, Y. Yang, J. W. Kwak, H. H. Jung, J. Song, C. Yang, A. Wang, Z. Liu, J. Y. Lee, B. H. Kim, J.-H. Kim, J. Lee, Y. Yu, B. J. Kim, H. Jang, K. J. Yu, J. Kim, J. W. Lee, J.-W. Jeong, Y. M. Song, Y. Huang, Y. Zhang, J. A. Rogers, Self-

assembled three dimensional network designs for soft electronics. *Nat. Commun.* **8**, 15894 (2017).

10. W. Ronan, V. S. Deshpande, N. A. Fleck, The tensile ductility of cellular Solids: The role of imperfections. *Int. J. Solids Struct.* **102-103**, 200–213 (2016).

11. A. J. D. Shaikeea, H. Cui, M. O'Masta, X. R. Zheng, V. S. Deshpande, The toughness of mechanical metamaterials. *Nat. Mater.* **21**, 297–304 (2022).

12. Y. Xiao, X. Hu, J. Wu, Z. Shen, S. Wang, S. Xu, J. Zhao, J. Chang, Y. Zhang, Imperfection-insensitive flexible random network materials with horseshoe microstructures. *J. Mech. Phys. Solids* **195**, 105968 (2025).

13. Q. Ma, H. Cheng, K.-I. Jang, H. Luan, K.-C. Hwang, J. A. Rogers, Y. Huang, Y. Zhang, A nonlinear mechanics model of bio-inspired hierarchical lattice materials consisting of horseshoe microstructures. *J. Mech. Phys. Solids* **90**, 179–202 (2016).

14. Y. Ma, X. Feng, J. A. Rogers, Y. Huang, Y. Zhang, Design and application of 'J-shaped' stress–Strain behavior in stretchable electronics: A review. *Lab Chip* **17**, 1689–1704 (2017).

15. D. B. Kolesky, K. A. Homan, M. A. Skylar-Scott, J. A. Lewis, Three-dimensional bioprinting of thick vascularized tissues. *Proc. Natl. Acad. Sci. U.S.A.* **113**, 3179–3184 (2016).

16. A. K. Miri, D. Nieto, L. Iglesias, H. Goodarzi Hosseinabadi, S. Maharjan, G. U. Ruiz-Esparza, P. Khoshakhlagh, A. Manbachi, M. R. Dokmeci, S. Chen, S. R. Shin, Y. S. Zhang, A. Khademhosseini, Microfluidics-enabled multimaterial maskless stereolithographic bioprinting. *Adv. Mater.* **30**, 1800242 (2018).

17. J. Seo, W. Y. Byun, F. Alisafaei, A. Georgescu, Y.-S. Yi, M. Massaro-Giordano, V. B. Shenoy, V. Lee, V. Y. Bunya, D. Huh, Multiscale reverse engineering of the human ocular surface. *Nat. Med.* **25**, 1310–1318 (2019).

18. X. Wang, R. Feiner, H. Luan, Q. Zhang, S. Zhao, Y. Zhang, M. Han, Y. Li, R. Sun, H. Wang, T.-L. Liu, X. Guo, H. Oved, N. Noor, A. Shapira, Y. Zhang, Y. Huang, T. Dvir, J. A. Rogers,

Three-dimensional electronic scaffolds for monitoring and regulation of multifunctional hybrid tissues. *Extreme Mech. Lett.* **35**, 100634 (2020).

19. H. M. A. Kolken, C. P. de Jonge, T. van der Sloten, A. F. Garcia, B. Pouran, K. Willemsen, H. Weinans, A. A. Zadpoor, Additively manufactured space-filling meta-implants. *Acta Biomater.* **125**, 345–357 (2021).

20. S. Cao, Y. Wei, R. Bo, X. Yun, S. Xu, Y. Guan, J. Zhao, Y. Lan, B. Zhang, Y. Xiong, T. Jin, Y. Lai, J. Chang, Q. Zhao, M. Wei, Y. Shao, Q. Quan, Y. Zhang, Inversely engineered biomimetic flexible network scaffolds for soft tissue regeneration. *Sci. Adv.* **9**, eadi8606 (2023).

21. H. Wang, J. Tian, Y. Jiang, S. Liu, J. Zheng, N. Li, G. Wang, F. Dong, J. Chen, Y. Xie, Y. Huang, X. Cai, X. Wang, W. Xiong, H. Qi, L. Yin, Y. Wang, X. Sheng, A 3D biomimetic optoelectronic scaffold repairs cranial defects. *Sci. Adv.* **9**, eabq7750 (2023).

22. J.-W. Jeong, G. Shin, S. I. Park, K. J. Yu, L. Xu, J. A. Rogers, Soft materials in neuroengineering for hard problems in neuroscience. *Neuron* **86**, 175–186 (2015).

23. X. Yang, T. Zhou, T. J. Zwang, G. Hong, Y. Zhao, R. D. Viveros, T.-M. Fu, T. Gao, C. M. Lieber, Bioinspired neuron-like electronics. *Nat. Mater.* **18**, 510–517 (2019).

24. M. Han, L. Chen, K. Aras, C. Liang, X. Chen, H. Zhao, K. Li, N. R. Faye, B. Sun, J.-H. Kim, W. Bai, Q. Yang, Y. Ma, W. Lu, E. Song, J. M. Baek, Y. Lee, C. Liu, J. B. Model, G. Yang, R. Ghaffari, Y. Huang, I. R. Efimov, J. A. Rogers, Catheter-integrated soft multilayer electronic arrays for multiplexed sensing and actuation during cardiac surgery. *Nat. Biomed. Eng* **4**, 997–1009 (2020).

25. B. Sadri, W. Gao, Fibrous wearable and implantable bioelectronics. *Appl. Phys. Rev.* **10**, 031303 (2023).

26. C. Yu, Y. Li, X. Zhang, X. Huang, V. Malyarchuk, S. Wang, Y. Shi, L. Gao, Y. Su, Y. Zhang, H. Xu, R. T. Hanlon, Y. Huang, J. A. Rogers, Adaptive optoelectronic camouflage systems with designs inspired by cephalopod skins. *Proc. Natl. Acad. Sci. U.S.A.* **111**, 12998–13003 (2014).

27. S. Choi, J. Park, W. Hyun, J. Kim, J. Kim, Y. B. Lee, C. Song, H. J. Hwang, J. H. Kim, T. Hyeon, D.-H. Kim, Stretchable heater using ligand-exchanged silver nanowire nanocomposite for wearable articular thermotherapy. *ACS Nano* **9**, 6626–6633 (2015).

28. S.-H. Byun, J. Y. Sim, Z. Zhou, J. Lee, R. Qazi, M. C. Walicki, K. E. Parker, M. P. Haney, S. H. Choi, A. Shon, G. B. Gereau, J. Bilbily, S. Li, Y. Liu, W.-H. Yeo, J. G. McCall, J. Xiao, J.-W. Jeong, Mechanically transformative electronics, sensors, and implantable devices. *Sci. Adv.* **5**, eaay0418 (2019).

29. K. Sim, Z. Rao, Z. Zou, F. Ershad, J. Lei, A. Thukral, J. Chen, Q.-A. Huang, J. Xiao, C. Yu, Metal oxide semiconductor nanomembrane-based soft unnoticeable multifunctional electronics for wearable human-machine interfaces. *Sci. Adv.* **5**, eaav9653 (2019).

30. Y. Wang, X. Li, S. Fan, X. Feng, K. Cao, Q. Ge, L. Gao, Y. Lu, Three-dimensional stretchable microelectronics by projection microstereolithography (PμSL). *ACS Appl. Mater. Interfaces* **13**, 8901–8908 (2021).

31. Y. Yu, J. Li, S. A. Solomon, J. Min, J. Tu, W. Guo, C. Xu, Y. Song, W. Gao, All-printed soft human-machine interface for robotic physicochemical sensing. *Sci. Robot.* **7**, eabn0495 (2022).

32. Z. Liu, X. Hu, R. Bo, Y. Yang, X. Cheng, W. Pang, Q. Liu, Y. Wang, S. Wang, S. Xu, Z. Shen, Y. Zhang, A three-dimensionally architected electronic skin mimicking human mechanosensation. *Science* **384**, 987–994 (2024).

33. J. Liu, D. Yan, W. Pang, Y. Zhang, Design, fabrication and applications of soft network materials. *Mater. Today* **49**, 324–350 (2021).

34. J. A. Lewis, G. M. Gratson, Direct writing in three dimensions. *Mater. Today* **7**, 32–39 (2004).

35. B. Derby, Printing and prototyping of tissues and scaffolds. *Science* **338**, 921–926 (2012).

36. R. L. Truby, J. A. Lewis, Printing soft matter in three dimensions. *Nature* **540**, 371–378 (2016).

37. L. R. Meza, S. Das, J. R. Greer, Strong, lightweight, and recoverable three-dimensional ceramic nanolattices. *Science* **345**, 1322–1326 (2014).

38. J. R. Tumbleston, D. Shirvanyants, N. Ermoshkin, R. Janusziewicz, A. R. Johnson, D. Kelly, K. Chen, R. Pinschmidt, J. P. Rolland, A. Ermoshkin, E. T. Samulski, J. M. DeSimone, Continuous liquid interface production of 3D objects. *Science* **347**, 1349–1352 (2015).

39. X. Zheng, W. Smith, J. Jackson, B. Moran, H. Cui, D. Chen, J. Ye, N. Fang, N. Rodriguez, T. Weisgraber, C. M. Spadaccini, Multiscale metallic metamaterials. *Nat. Mater.* **15**, 1100–1106 (2016).

40. S. Xu, Z. Yan, K.-I. Jang, W. Huang, H. Fu, J. Kim, Z. Wei, M. Flavin, J. McCracken, R. Wang, A. Badea, Y. Liu, D. Xiao, G. Zhou, J. Lee, H. U. Chung, H. Cheng, W. Ren, A. Banks, X. Li, U. Paik, R. G. Nuzzo, Y. Huang, Y. Zhang, J. A. Rogers, Materials science. Assembly of micro/nanomaterials into complex, three-dimensional architectures by compressive buckling. *Science* **347**, 154–159 (2015).

41. Y. Zhang, F. Zhang, Z. Yan, Q. Ma, X. Li, Y. Huang, J. A. Rogers, Printing, folding and assembly methods for forming 3D mesostructures in advanced materials. *Nat. Rev. Mater.* **2**, 17019 (2017).

42. H. Fu, K. Nan, W. Bai, W. Huang, K. Bai, L. Lu, C. Zhou, Y. Liu, F. Liu, J. Wang, M. Han, Z. Yan, H. Luan, Y. Zhang, Y. Zhang, J. Zhao, X. Cheng, M. Li, J. W. Lee, Y. Liu, D. Fang, X. Li, Y. Huang, Y. Zhang, J. A. Rogers, Morphable 3D mesostructures and microelectronic devices by multistable buckling mechanics. *Nat. Mater.* **17**, 268–276 (2018).

43. X. Cheng, Z. Fan, S. Yao, T. Jin, Z. Lv, Y. Lan, R. Bo, Y. Chen, F. Zhang, Z. Shen, H. Wan, Y. Huang, Y. Zhang, Programming 3D curved mesosurfaces using microlattice designs. *Science* **379**, 1225–1232 (2023).

44. R. Bo, S. Xu, Y. Yang, Y. Zhang, Mechanically-guided 3D assembly for architected flexible electronics. *Chem. Rev.* **123**, 11137–11189 (2023).

45. Z. Yan, F. Zhang, F. Liu, M. Han, D. Ou, Y. Liu, Q. Lin, X. Guo, H. Fu, Z. Xie, M. Gao, Y. Huang, J. Kim, Y. Qiu, K. Nan, J. Kim, P. Gutruf, H. Luo, A. Zhao, K.-C. Hwang, Y. Huang, Y. Zhang, J. A. Rogers, Mechanical assembly of complex, 3D mesostructures from releasable multilayers of advanced materials. *Sci. Adv.* **2**, e1601014 (2016).

46. X. Cheng, Z. Liu, T. Jin, F. Zhang, H. Zhang, Y. Zhang, Bioinspired design and assembly of a multilayer cage-shaped sensor capable of multistage load bearing and collapse prevention. *Nanotechnology* **32**, 155506 (2021).

47. X. Guo, X. Wang, D. Ou, J. Ye, W. Pang, Y. Huang, J. A. Rogers, Y. Zhang, Controlled mechanical assembly of complex 3D mesostructures and strain sensors by tensile buckling. *NPJ Flex. Electron.* **2**, 14 (2018).

48. S. An, Y. Cao, H. Jiang, A mechanically robust and facile shape morphing using tensile-induced buckling. *Sci. Adv.* **10**, eado8431 (2024).

49. A. Rafsanjani, L. Jin, B. Deng, K. Bertoldi, Propagation of pop ups in kirigami shells. *Proc. Natl. Acad. Sci. U.S.A.* **116**, 8200–8205 (2019).

50. S. Babaee, Y. Shi, S. Abbasalizadeh, S. Tamang, K. Hess, J. E. Collins, K. Ishida, A. Lopes, M. Williams, M. Albaghdadi, A. M. Hayward, G. Traverso, Kirigami-inspired stents for sustained local delivery of therapeutics. *Nat. Mater.* **20**, 1085–1092 (2021).

51. W. Huang, T. Yu, K. J. Hsia, S. Adriaenssens, M. Liu, Integration of kinks and creases enables tunable folding in meta-ribbons. *Matter* **7**, 3007–3023 (2024).

52. W. Huang, T. Yu, D. Vella, K. J. Hsia, M. Liu, Exploiting dynamic bifurcation in elastic ribbons for mode skipping and selection. *J. Mech. Phys. Solids* **190**, 105721 (2024).

53. K. Guo, M. Suñé, M. L. Kwok, K. J. Hsia, M. Liu, D. Vella, Localized tension–induced giant folding in unstructured elastic sheets. *Proc. Natl. Acad. Sci. U.S.A.* **122**, e2423439122 (2025).

54. W. Huang, Z. Hao, J. Li, D. Tong, K. Guo, Y. Zhang, H. Gao, K. J. Hsia, M. Liu, A tutorial on simulating nonlinear behaviors of flexible structures with the discrete differential geometry (DDG) method. *Appl. Mech. Rev.*, 1–88 (2025).

55. F. Fernandez, M. A. Puso, J. Solberg, D. A. Tortorelli, Topology optimization of multiple deformable bodies in contact with large deformations. *Comput. Methods Appl. Mech. Eng.* **371**, 113288 (2020).

56. X. S. Zhang, H. Chi, G. H. Paulino, Adaptive multi-material topology optimization with hyperelastic materials under large deformations: A virtual element approach. *Comput. Methods Appl. Mech. Eng.* **370**, 112976 (2020).

57. X. Chen, S. Yao, J. Yvonnet, Nonlinear topology optimization of flexoelectric soft dielectrics at large deformation. *Comput. Methods Appl. Mech. Eng.* **427**, 117005 (2024).

58. Z. Du, Y. Guo, C. Liu, W. Zhang, R. Xue, Y. Guo, S. Tang, X. Guo, Structural topology optimization of three-dimensional multi-material composite structures with finite deformation. *Compos. Struct.* **328**, 117692 (2024).

59. G. X. Gu, C.-T. Chen, D. J. Richmond, M. J. Buehler, Bioinspired hierarchical composite design using machine learning: Simulation, additive manufacturing, and experiment. *Mater. Horizons* **5**, 939–945 (2018).

60. L. Wu, L. Liu, Y. Wang, Z. Zhai, H. Zhuang, D. Krishnaraju, Q. Wang, H. Jiang, A machine learning-based method to design modular metamaterials. *Extreme Mech. Lett.* **36**, 100657 (2020).

61. S. Lee, Z. Zhang, G. X. Gu, Generative machine learning algorithm for lattice structures with superior mechanical properties. *Mater. Horizons* **9**, 952–960 (2022).

62. R. van Mastrigt, M. Dijkstra, M. van Hecke, C. Coulais, Machine learning of implicit combinatorial rules in mechanical metamaterials. *Phys. Rev. Lett.* **129**, 198003 (2022).

63. C. S. Ha, D. Yao, Z. Xu, C. Liu, H. Liu, D. Elkins, M. Kile, V. Deshpande, Z. Kong, M. Bauchy, X. Zheng, Rapid inverse design of metamaterials based on prescribed mechanical behavior through machine learning. *Nat. Commun.* **14**, 5765 (2023).

64. T. Jin, X. Cheng, S. Xu, Y. Lai, Y. Zhang, Deep learning aided inverse design of the buckling-guided assembly for 3D frame structures. *J. Mech. Phys. Solids* **179**, 105398 (2023).

65. Y. Jia, K. Liu, X. S. Zhang, Topology optimization of irregular multiscale structures with tunable responses using a virtual growth rule. *Comput. Methods Appl. Mech. Eng.* **425**, 116864 (2024).

66. Y. Jia, K. Liu, X. S. Zhang, Modulate stress distribution with bio-inspired irregular architected materials towards optimal tissue support. *Nat. Commun.* **15**, 4072 (2024).

67. X. Lei, C. Liu, Z. Du, W. Zhang, X. Guo, Machine learning-driven real-time topology optimization under moving morphable component-based framework. *J. Appl. Mech. Trans. ASME* **86**, 011004 (2018).

68. D. Liu, H. Yang, K. I. Elkhodary, S. Tang, X. Guo, Cyclic softening in nonlocal shells—A data-driven graph-gradient plasticity approach. *Extreme Mech. Lett.* **60**, 101995 (2023).

69. J. O. V. Delgadillo, S. Delorme, F. Thibault, R. DiRaddo, S. G. Hatzikiriakos, Large deformation characterization of porcine thoracic aortas: Inverse modeling fitting of uniaxial and biaxial tests. *J. Biomed. Eng.* **8**, 717 (2015).

70. L. Onural, F. Yaraş, H. Kang, Digital holographic three-dimensional video displays. *Proc. IEEE* **99**, 576–589 (2011).

71. J. An, K. Won, Y. Kim, J.-Y. Hong, H. Kim, Y. Kim, H. Song, C. Choi, Y. Kim, J. Seo, A. Morozov, H. Park, S. Hong, S. Hwang, K. Kim, H.-S. Lee, Slim-panel holographic video display. *Nat. Commun.* **11**, 5568 (2020).

72. X. Xia, Z. Zheng, X. Liu, H. Li, C. Yan, Omnidirectional-view three-dimensional display system based on cylindrical selective-diffusing screen. *Appl. Optics* **49**, 4915–4920 (2010).

73. L. Ni, Z. Li, H. Li, X. Liu, 360-degree large-scale multiprojection light-field 3D display system. *Appl. Optics* **57**, 1817–1823 (2018).

74. D. E. Smalley, E. Nygaard, K. Squire, J. Van Wagoner, J. Rasmussen, S. Gneiting, K. Qaderi, J. Goodsell, W. Rogers, M. Lindsey, K. Costner, A. Monk, M. Pearson, B. Haymore, J. Peatross, A photophoretic-trap volumetric display. *Nature* **553**, 486–490 (2018).

75. R. Hirayama, D. Martinez Plasencia, N. Masuda, S. Subramanian, A volumetric display for visual, tactile and audio presentation using acoustic trapping. *Nature* **575**, 320–323 (2019).

76. D. C. Kim, H. Yun, J. Kim, H. Seung, W. S. Yu, J. H. Koo, J. Yang, J. H. Kim, T. Hyeon, D.-H. Kim, Three-dimensional foldable quantum dot light-emitting diodes. *Nat. Electron.* **4**, 671–680 (2021).

77. D. Lanman, M. Hirsch, Y. Kim, R. Raskar, Content-adaptive parallax barriers: Optimizing dual-layer 3D displays using low-rank light field factorization. *ACM Trans. Graph.* **29**, 163 (2010).

78. R. Yang, X. Huang, S. Li, C. Jaynes, Toward the light field display: Autostereoscopic rendering via a cluster of projectors. *IEEE Trans. Vis. Comput. Graph.* **14**, 84–96 (2008).

79. H. Arimoto, B. Javidi, Integral three-dimensional imaging with digital reconstruction. *Opt. Lett.* **26**, 157–159 (2001).

80. G. E. Favalora, Volumetric 3D displays and application infrastructure. *Computertomographie* **38**, 37–44 (2005).

81. N. S. Holliman, N. A. Dodgson, G. E. Favalora, L. Pockett, Three-dimensional displays: A review and applications analysis. *IEEE Trans. Broadcast.* **57**, 362–371 (2011).
